# Specific Photocatalytic C–C Coupling of Benzyl Alcohol to Deoxybenzoin or Benzoin by Precise Control of C_α_–H Bond Activation or O–H Bond Activation by Adjusting the Adsorption Orientation of Hydrobenzoin Intermediates

**DOI:** 10.1021/acscatal.4c03426

**Published:** 2024-10-02

**Authors:** Zongyang Yue, Guanchu Lu, Wenjing Wei, Yanan Deng, Luxi Yang, Shibo Shao, Xianfeng Chen, Yi Huang, Jianhua Qian, Xianfeng Fan

**Affiliations:** † Institute for Materials and Processes, School of Engineering, The University of Edinburgh, Edinburgh EH9 3BF, U.K.; ‡ Petrochemical Research Institute, PetroChina Company Limited, Beijing 102206, China; § Institute for Bioengineering, School of Engineering, The University of Edinburgh, Edinburgh EH9 3BF, U.K.; ∥ School of Petrochemical Engineering, Liaoning Petrochemical University, Fushun 113001, China

**Keywords:** C−C coupling of benzyl alcohol, C_α_−H bond activation, O−H bond activation, photocatalysis, cadmium sulfide

## Abstract

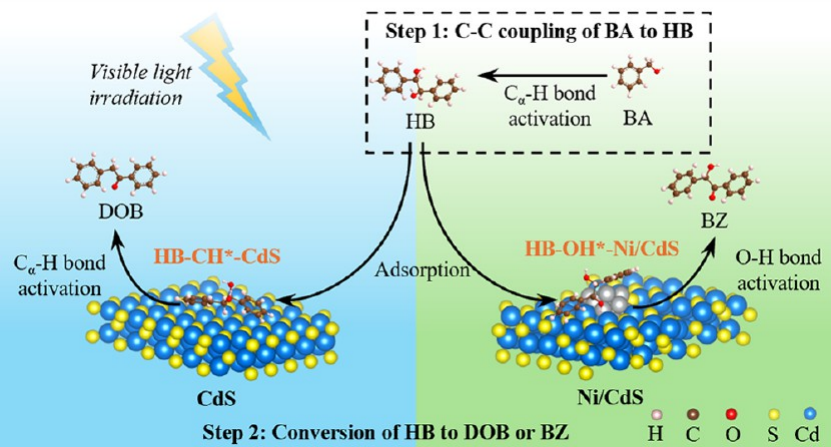

Benzyl alcohol (BA) is a major biomass derivative and can be further converted into deoxybenzoin (DOB) and benzoin (BZ) as high-value products for industrial applications through photocatalytic C–C coupling reaction. The photocatalytic process contains two reaction steps, which are (1) the C–C coupling of BA to hydrobenzoin (HB) intermediates and (2) either dehydration of HB to DOB or dehydrogenation of HB to BZ. We found that generation of DOB or BZ is mainly determined by the activation of C_α_–H or O–H bonds in HB. In this study, phase junction CdS photocatalysts and Ni/CdS photocatalysts were elaborately designed to precisely control the activation of C_α_–H or O–H bonds in HB by adjusting the adsorption orientation of HB on the photocatalyst surfaces. After orienting the C_α_–H groups in HB on the CdS surfaces, the C_α_–H bond dissociation energy (BDE) at 1.39 eV is lower than the BDE of the O–H bond at 2.69 eV, therefore improving the selectivity of the DOB. Conversely, on Ni/CdS photocatalysts, the O–H groups in HB orient toward the photocatalyst surfaces. The BDE of the O–H bonds is 1.11 eV to form BZ, which is lower than the BDE of the C_α_–H bonds to the DOB (1.33 eV), thereby enhancing the selectivity of BZ. As a result, CdS photocatalysts can achieve complete conversion of BA to 80.4% of the DOB after 9 h of visible light irradiation, while 0.3% Ni/CdS photocatalysts promote complete conversion of BA to 81.5% of BZ after only 5 h. This work provides a promising strategy in selective conversion of BA to either DOB or BZ through delicate design of photocatalysts.

## 1 Introduction

Benzyl alcohol (BA) is a promising biomass derivative and can be further converted into high-value products for industrial applications, such as deoxybenzoin (DOB) as the flame retardant in the polymer industry,^1,2^ and benzoin (BZ) for surface coatings and photosensitive polymer materials.^3−5^ However, traditional methods for generating DOB and BZ, such as *N*-heterocyclic carbene (NHC)-catalyzed BZ condensation^3,6−8^ and Friedel–Crafts acylation,^9,10^ respectively, show several significant drawbacks. First, these methods require the use of activators, such as diazabicycloundecane (DBU), HCl, NaOH, or TEA for the NHC-catalyzed process^6,8^ and K_2_CO_3_, KF, K_3_PO_4_, or KOAc in Friedel–Crafts acylation,^10^ which increase the production costs. Second, the recycling and reusability of these catalysts are limited, with significant decreases in the yield of products after several cycles.^6−8^ Third, the preparation of NHC catalysts is particularly complex and costly, requiring expensive palladium or copper catalysts along with BINAP and X-Phos ligands for the introduction of alkyl or aryl groups at specific N positions in the NHC precursors.^11,12^ These drawbacks limit the applications of these traditional strategies for generation of DOB and BZ. Photocatalytic C–C coupling of BA to DOB and BZ is an efficient and cost-effective alternative, overcoming the need for additional activators, simplifying catalyst preparation, and enhancing photocatalyst reusability.

**Scheme 1:**
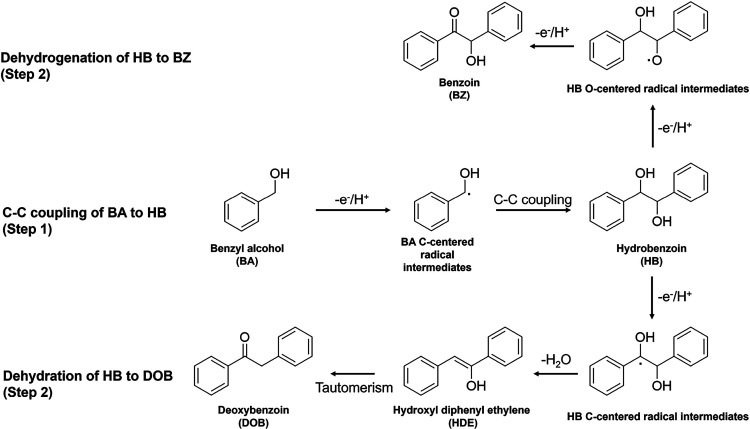
Photocatalytic C–C Coupling of BA to DOB and BZ Reproduced from refs (13) and (14). Copyright [2020] American Chemical Society.

Previous works have demonstrated that the generation of DOB and BZ through photocatalytic C–C coupling of BA is a two-step reaction process.^13−15^ As shown in Scheme 1, the C_α_–H bonds in BA are first activated to form BA C-centered radical intermediates and then convert to hydrobenzoin (HB) as an intermediate product through self-C–C coupling reaction (Step 1).^13−15^ In Step 2, HB intermediates are further converted to either DOB through dehydration of HB or BZ through dehydrogenation of HB. In these reactions, the selectivity of DOB and BZ is mainly controlled by the competition between the dehydration and dehydrogenation of HB intermediates.^13−15^ Previous works have demonstrated that the dehydration of HB intermediates to DOB is mainly determined by the C_α_–H bond activation.^14^ Wang’s group has demonstrated that the activation of C_α_–H bonds in HB to generate HB C-centered radical intermediates by ZnIn sulfide photocatalysts is an important step, as the intermediates can be readily reduced to hydroxyl diphenyl ethylene (HDE) by photoexcited electrons and then generate DOB through HDE tautomerism.^14^ However, the mechanism in the dehydrogenation of HB intermediates to BZ is still unclear. Generally, the dehydrogenation of alcoholic compounds to corresponding products, such as dehydrogenation of BA to BH and methanol to formaldehyde, is mainly determined by the O–H bond activation.^16−19^ For example, in the dehydrogenation of methanol into formaldehyde, the activation of O–H bonds in methanol to generate CH_3_O• radical intermediates can significantly reduce the bond dissociation energy (BDE) of C–H bonds from 1.57 eV in methanol to 0.21 eV, thereby enhancing formaldehyde production.^20−22^ Inspired by these works, the selectivity in the generation of DOB or BZ from C–C coupling of BA should be precisely controlled by the activation of either C_α_–H bonds or O–H bonds in HB intermediates, respectively.

Based on the above discussions, it is important to design photocatalysts that can precisely control the activation of either the C_α_–H bonds or the O–H bonds in HB intermediates to improve the selectivity of either DOB or BZ as desirable products, respectively. Metal sulfide photocatalysts show promising potential in the activation of the C_α_–H bonds in alcoholic compounds to generate corresponding products, such as C–C coupling of methane to ethylene glycol and C–C coupling of furfuryl alcohol (FA) to hydrofuroin (HF). The sulfur moieties on the surfaces of the metal sulfide photocatalysts exhibit high activity for the C_α_–H bond activation in these alcoholic compounds.^22−24^ For example, Wang’s group demonstrated that the sulfur moieties on the CdS surfaces can improve the activation of C_α_–H bonds in methanol to form •CH_2_OH instead of activation of O–H bonds to form CH_3_O•, as the sulfur moieties on CdS surfaces act as proton acceptors, significantly reducing the reaction energy of C_α_–H bond activation to 0.8 eV compared to 1.6 eV for O–H bond activation, therefore improving the generation of ethylene glycol.^22^ Amal’s group has demonstrated that the sulfur moieties on the Zn_*x*_In_2_S_3+*x*_ photocatalysts can accumulate the photogenerated holes to form a sulfur anion radical (S^–•^). S^–•^ can promote the concerted proton–electron transfer (CPET) pathway and improve the C_α_–H bond activation in FA, therefore improving the generation of HF.^24^ Herein, the elaborately designed CdS photocatalysts with exposed sulfur moieties might have a high potential to improve the C_α_–H bond activation in HB intermediates and enhance the selectivity of DOB as a desirable product.

Activation of the O–H bonds in HB intermediates can enhance the generation of BZ as a desirable product from C–C coupling of BA. Generally, metal clusters, including Pt, Au, Ru, and Ni, have been proved as cocatalysts to improve the activation of the O–H bonds in alcoholic compounds in the generation of dehydrogenated products, such as dehydrogenation of BA to BH and dehydrogenation of methanol to formaldehyde. These metal clusters can improve the adsorption of O atoms in O–H groups on the photocatalyst surfaces, therefore enhancing the activation of O–H bonds in alcoholic compounds.^16,25−27^ For example, Li’s group demonstrated that the introduction of Ru clusters on the surfaces of g-C_3_N_4–*x*_ photocatalysts can decrease the adsorption energy of BA from −0.024 eV without Ru clusters to −1.542 eV and elongate the O–H bonds in BA from 0.994 to 1.011 Å, therefore enhancing the selectivity of BH as the dehydrogenated product.^27^ Xu’s work showed that Ni clusters on CdS nanoparticles act as an electron acceptor, improving the activation of O–H bonds in absorbed alcohols to form O-centered radical intermediates, which then oxidize into corresponding dehydrogenated ketone compounds.^16^ Inspired by the above research, the introduction of metal clusters as cocatalysts on the photocatalysts might have potential in improving the activation of the O–H bonds in HB intermediates to enhance the selectivity of BZ as a desirable product.

In summary, the generation of DOB and BZ from C–C coupling of BA contains two reaction steps: (1) the C–C coupling of BA to HB intermediates and (2) the conversion of HB intermediates into either DOB through a dehydration process or into BZ through a dehydrogenation process. Activations of C_α_–H bonds and O–H bonds in HB intermediates are the determined steps to control the selectivity of DOB and BZ as desirable products, respectively. In this study, phase junction CdS photocatalysts (CdS) and decoration of Ni clusters on the surfaces of phase junction CdS photocatalysts (Ni/CdS) were designed to improve the C_α_–H bond activation and the activation of the O–H bond in HB, respectively. In detail, CdS photocatalysts adsorb the HB intermediates with an orientation of the C_α_–H groups toward the CdS surfaces (R-CH*). This orientation significantly reduces the energy for C_α_–H bond activation in HB in comparison to that of the activation of the O–H bond, therefore enhancing the selectivity of the DOB. Conversely, the Ni/CdS photocatalysts preferentially adsorb the HB intermediates with orientation of the O–H groups toward its surfaces (R-OH*), which dramatically improves the activation of the O–H bond in HB, therefore improving the selectivity of BZ. Our experimental results demonstrated that phase junction CdS photocatalysts can achieve complete conversion of BA to 80.4% of DOB after 9 h of visible light irradiation, while 0.3% Ni/CdS photocatalysts show the complete conversion of BA to 81.5% of BZ after only 5 h of visible light irradiation, which exhibit the best photocatalytic performance compared to previous works.

## 2 Materials and Methods

### 2.1 Materials

All reagents were of analytical grade and were used without further purification. Trisodium citrate dihydrate (99%+), thioacetamide (99%+), CH_3_CN, BZ, and NiCl_2_·4H_2_O (98%) were purchased from Fisher Scientific International, Inc. Methanol (MEOH), 5,5-dimethyl-1-pyrroline *N*-oxide (DMPO), Cd(NO_3_)_2_·4H_2_O (98%), BA, BH, DOB, HB, 1,1-diphenylethylene (DPE), furfuryl alcohol (FA), methylbenzyl alcohol (Me-BA), methoxybenzyl alcohol (MeO-BA), Na_2_S·9H_2_O (98%+), Na_2_S_2_O_8_ (99%), and Na_2_SO_3_ (98.5%) were purchased from Sigma-Aldrich Co., Ltd. Ethylene glycol (EG) was purchased from VWR International, LLC. Deionized (DI) water was produced by CENTRA R200 Centralized Purification and Distribution Systems.

### 2.2 Preparation of Phase Junction CdS Nanoparticles

The phase junction CdS photocatalysts (CdS) were prepared by a one-pot hydrothermal method. Typically, 308 mg of Cd(NO_3_)_2_·4H_2_O and 150 mg of trisodium citrate were dissolved into a 15 mL water and EG mixed solution (v_water_/v_EG_ = 1/5). After ultrasonic dispersion for 10 min and then vigorous stirring for 30 min, 375 mg of thioacetamide was added into the solution. After being stirred for another 30 min, the mixture was transferred into a 25 mL stainless Teflon-lined autoclave reactor. The autoclave reactor was subsequently heated to 160 °C with a 3 °C min^–1^ heating rate in an oven, and the temperature was maintained for 4 h. After being naturally cooled down, the sample was collected by centrifugation (9000 rpm) and rinsed several times with ethanol and water. The solid samples were then dried under vacuum at 60 °C for 4 h.

### 2.3 Preparation of Ni/CdS

The Ni/CdS photocatalysts were formed from CdS and NiCl_2_ through in situ photocatalytic synthesis. Typically, 10 mg of CdS nanoparticles was dispersed in a 5 mL CH_3_CN solution containing 10 mg of BA and *x*% of NiCl_2_ (*x*% = molar of NiCl_2_/molar of 10 mg CdS; 0.1, 0.3, and 0.5%). The mixed solution was stirred, and argon was purged in the sealed reactor for 30 min. Then, the sealed reactor was illuminated with a xenon arc lamp with a 420 nm UV filter. During the irradiation stage, the temperature of the solution was kept at 20 °C with cooling water. After the irradiation, the sample was collected by centrifugation (9000 rpm) and rinsed several times with CH_3_CN. The solid samples were dried under vacuum at 60 °C for 4 h and stored under an inert atmosphere condition.

### 2.4 Photocatalytic Activity Evaluation

Photocatalytic reactions were performed in a customized quartz reactor with a cooling water jacket. As shown in Figure S1, the visible light source was provided by a xenon arc lamp (Perfect Light Company, PE300BF) equipped with a 420 nm UV filter. The position of the lamp was fixed during the entire experiment to maintain a constant light intensity of 0.35 W cm^–2^. A 10 mg portion of reactants (BA, MeO-BA, Me-BA, and FA) and 10 mg of CdS photocatalysts were dispersed in 5 mL of CH_3_CN in the reactor with a certain amount of NiCl_2_ (*x*% = molar of NiCl_2_/molar of 10 mg of CdS; 0, 0.1, 0.3, and 0.5%). Cooling water was employed to keep the temperature of reaction solution at 20 °C. The photocatalysts were dispersed by magnetic stirring, and the reactor was firmly sealed after 30 min of argon purge (10 mL min^–1^). The sealed reactor with 200 rpm of magnetic stirring was illuminated under visible light irradiation. After the reaction, the solution was collected by centrifugation and methylparaben was added as the internal standard. The obtained solution was qualitatively analyzed by gas chromatography–mass spectrometry (GC-MS, QP2010 SE, Shimadzu, Rxi-5 ms column) and quantitatively analyzed by gas chromatography (GC, GC-2010 plus, Shimadzu, Stabilwax-MS column). The following equations were used to calculate the conversion rate of reactants, the selectivity of products, and the yield of products:
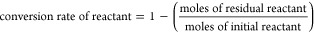

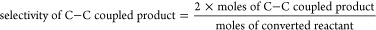

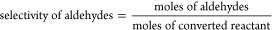

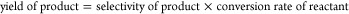


### 2.5 Characterization

The morphologies of as-prepared CdS and 0.3% Ni/CdS were imaged by high-resolution transmission electron microscopy (TEM/HRTEM, JEOL JEM-2100F). The composition of *x*% Ni/CdS photocatalysts was determined by inductively coupled plasma optical emission spectroscopy (ICP-OES, Varian Vista Pro). The chemical states of each element in *x*% Ni/CdS photocatalysts and X-ray photoelectron valence band spectra (XPS-VB) were characterized by X-ray photoelectron spectroscopy (XPS, ThermoFisher K-Alpha) equipped with an Al Kα X-ray source. All peaks have been calibrated with the C 1s peak, where the standard binding energy (B.E.) is 284.8 eV for the adventitious carbon source. X-ray diffraction (XRD) patterns were obtained using a Bruker Phaser-D2 diffractometer with a Cu Kα X-ray source at the voltage of 40 kV and the current of 40 mA. The light absorption properties and bandgap results of prepared *x*% Ni/CdS were obtained using UV/vis diffuse reflectance spectroscopy (DRS) on a JASCO V-670 spectrophotometer equipped with an integration sphere in the spectral range of 200–1000 nm and BaSO_4_ was used as the reflectance standard. The photoelectrochemical (PEC) measurements were performed in a standard three-electrode system by using an electrochemical workstation (CHI660E, Chenhua, shanghai) under visible light illumination in 50 mL of CH_3_CN with 30 mg of BA and 0.2 M Na_2_ClO_4_. Photoluminescence (PL) analyses were conducted on a Shimadzu RF-6000. The time-resolved photoluminescence (TRPL) experiments were conducted on an Edinburgh FLS1000. More detailed characterization information is provided in the Supporting Information.

## 3 Results and Discussion

### 3.1 Characterization of Phase Junction CdS and Ni/CdS Photocatalysts

**Figure 1:**
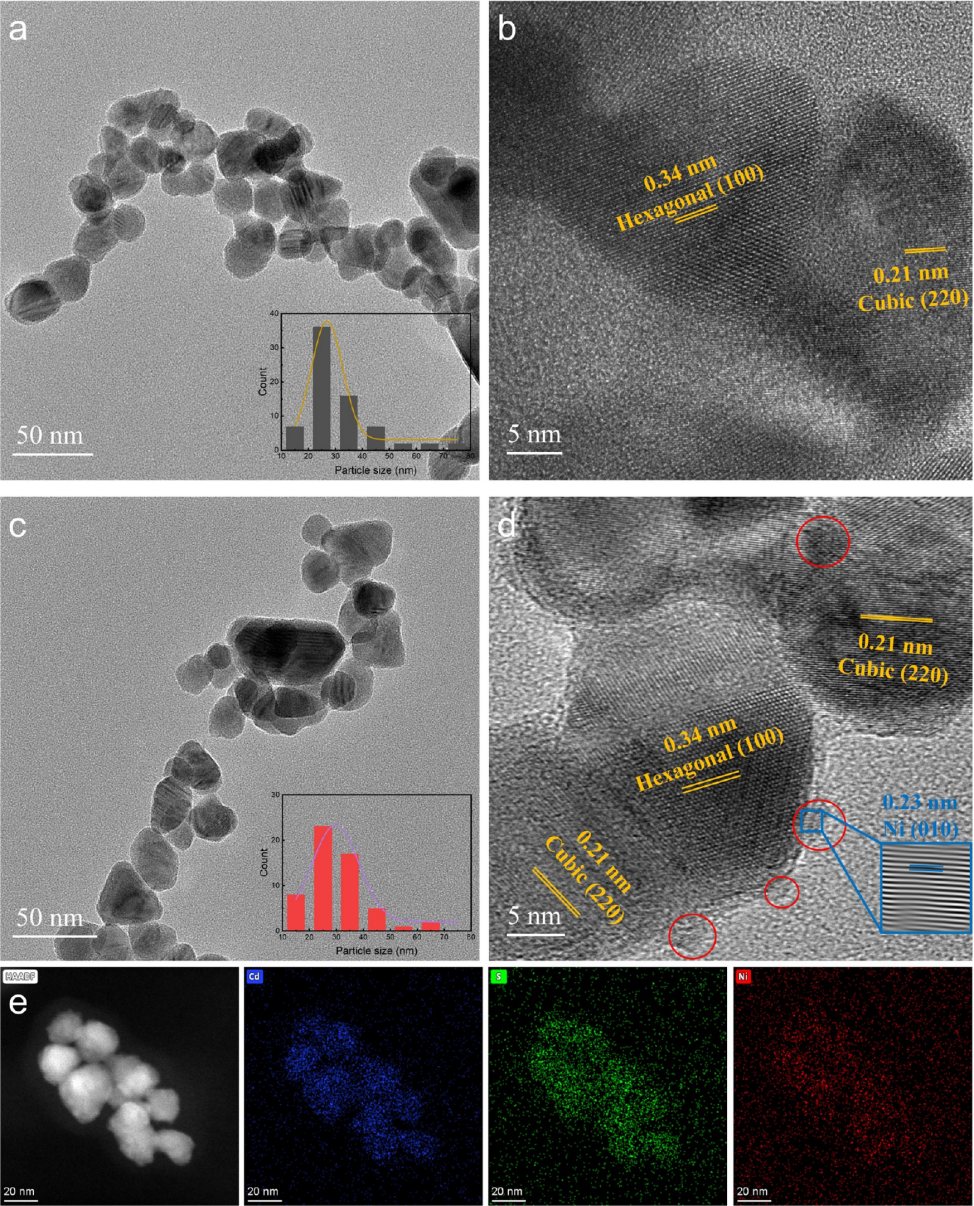
TEM and HRTEM images of phase junction CdS (a, b) and 0.3% Ni/CdS (c, d). (e) HAADF-STEM images and corresponding elemental mappings of 0.3% Ni/CdS photocatalysts. The inset graphs in panels (a) and (c) are corresponding particle size distributions.

TEM/HRTEM, XRD, ICP-OES, and XPS were conducted to verify the successful synthesis of phase junction CdS nanoparticles (CdS) and the loading of Ni clusters onto phase junction CdS nanoparticles (Ni/CdS). The phase junction CdS photocatalysts were prepared through the hydrothermal process reported in our previous work,^28^ while the Ni/CdS photocatalysts were prepared through an in situ photodeposition process using NiCl_2_ as the precursor under visible light irradiation. As shown in Figure 1a,c, TEM images display comparable irregular nanoparticles with an average particle size of around 30 nm for both CdS and 0.3% Ni/CdS photocatalysts. The TEM element mappings (Figure 1e and Figure S2) show Ni uniformly dispersed on the surfaces of the CdS nanoparticles. HRTEM images (Figure 1b,d) confirm the presence of hexagonal CdS (100) and cubic CdS (220) facets in both CdS and 0.3% Ni/CdS photocatalysts. Additionally, the 0.3% Ni/CdS photocatalysts exhibit a lattice spacing of 0.23 nm (red circle), corresponding to the (010) plane of metallic Ni.^29^

Figure S3 presents the XRD patterns of CdS and *x*% Ni/CdS and the standard patterns of CdS (hexagonal phase of CdS: JCPDS no. 41-1049; cubic phase of CdS: JCPDS no. 10-0454). These results exhibit the presence of both hexagonal CdS phase and cubic CdS phase in CdS and *x*% Ni/CdS. Upon decorating CdS with Ni clusters, there are no obvious changes in the XRD patterns of CdS, indicating that the photodeposition of Ni onto CdS does not alter the structure of CdS.

XPS was conducted to further investigate the chemical states of CdS and 0.3% Ni/CdS photocatalysts. The full XPS spectra are presented in Figure S4a and demonstrate that both CdS and 0.3% Ni/CdS photocatalysts exhibit peaks of Cd and S elements, and 0.3% Ni/CdS additionally displays peaks of the Ni element. Figure S4b,c demonstrates the high-resolution XPS spectra of Cd and S elements in CdS and 0.3% Ni/CdS photocatalysts, respectively. In CdS, the XPS S 2p peaks are observed at 161.5 eV (S 2p_3/2_) and 162.8 eV (S 2p_1/2_), whereas in 0.3% Ni/CdS, the bonding energy of these peaks shows the positive shift to 161.8 eV (S 2p_3/2_) and 163.1 eV (S 2p_1/2_), respectively. Similarly, the Cd 3d peaks in CdS are located at 405.1 eV (Cd 3d_5/2_) and 411.9 eV (Cd 3d_3/2_), whereas in 0.3% Ni/CdS, the binding energy of these peaks exhibits the positive shift to 405.5 eV (Cd 3d_5/2_) and 412.2 eV (Cd 3d_3/2_), respectively. Generally, the binding energy of an element originates from the Coulomb attraction between the electrons of atom and the atomic nucleus.^15,30^ A higher binding energy of an element indicates a decreased electron density, as the reduction in electron density enhances the Coulomb attraction between the remaining electrons and the atomic nucleus.^15,30^ The positive shifts of both S 2p and Cd 3d from CdS to 0.3% Ni/CdS exhibit the decreased electron density of S 2p and Cd 3d in 0.3% Ni/CdS, as the electrons transfer from CdS to Ni. As shown in Figure S4d, the high-resolution XPS spectrum of the Ni element in 0.3% Ni/CdS reveals the presence of metallic Ni, evidenced by a peak at 852.4 eV. In addition, three fitted peaks are also observed at 855.9 eV (Ni 2p_3/2_), 860.7 eV (Ni 2p_3/2_), and 873 eV (Ni 2p_1/2_), corresponding to the oxidation of Ni to Ni^2+^.^31^ The oxidation of Ni to Ni^2+^ is attributed to the inevitable oxidation occurring during the transportation stage of the samples for XPS characterization.

ICP-OES was conducted to measure the actual content of the Ni element in *x*% Ni/CdS. As shown in Table S1, the ratio of Ni/Cd in *x*% Ni/CdS increases from 0 in CdS to 0.43% in 0.5% Ni/CdS. The results indicate that the concentration of Ni clusters on the surfaces of CdS proportionally increases with the amount of NiCl_2_ added in the in situ photodeposition process, closely matching the theoretical amount of Ni in *x*% Ni/CdS photocatalysts. In summary, all characterizations prove that phase junction CdS photocatalysts and Ni/CdS photocatalysts were successfully prepared.

### 3.2 C–C Coupling of BA to DOB and BZ Using CdS and Ni/CdS Photocatalysts

After the structural characteristics of CdS and Ni/CdS photocatalysts were understood, both photocatalysts were employed to facilitate the C–C coupling of BA to DOB and BZ as desirable products. The mass spectrometry data from GC-MS, GC spectra, and ^1^H and ^13^C NMR spectra were collected to investigate the generated products in the photocatalytic C–C coupling of BA. As shown in Figure S5, the mass spectra of generated products along with the corresponding standard mass spectra from the GC-MS library confirm that the generated products were BH, HB, DOB, and BZ. As shown in Figure S6, the GC spectra demonstrate that BZ is the main product when using 0.3% Ni/CdS photocatalysts after 5 h of visible light irradiation, whereas DOB is the major product when using CdS photocatalysts after 9 h of reaction. In addition, ^1^H and ^13^C NMR analyses have been applied to BA, all products, and the reaction solutions after visible light irradiation. The results indicate that the main product is BZ when using 0.3% Ni/CdS and DOB when using CdS (the detailed discussions are presented in Figure S7).

This reaction contains two reaction steps, as shown in Scheme 1. In the first reaction step, BA as a reactant can convert to HB as an intermediate product through self-C–C coupling reaction, alongside with production of BH as a byproduct. In the second reaction step, HB intermediates can either dehydrogenate to BZ or dehydrate to DOB. Generally, both conversion of BA to BH and dehydrogenation of HB to BZ are net oxidation reactions that primarily rely on photogenerated holes in the photocatalytic system.^14^ In contrast, the C–C coupling of BA to HB and the dehydration of HB to DOB require both reductive and oxidative capabilities,^14^ needing both photogenerated holes and electrons. In this reaction step, DOB and BZ might have the potential to interconvert. To verify this possibility, DOB and BZ are separately introduced as a reactant into the reaction system, revealing their nonconvertibility (entries 1 and 2 in Table 1). Previous works demonstrated that BH can be converted to HB intermediates through C_α_=O bond activation.^32^ To exclude this possibility, BH was added as the reactant in our reaction system, and the results demonstrate that BH is an ultimate byproduct and cannot be converted to HB intermediates (entry 3 in Table 1).

**Table 1:** Controlled Reaction Conditions for C–C Coupling of BA to DOB and BZ^a^

				conversion/selectivity				
entry	photocatalyst	atmosphere	reactant	BA	BH	DOB	BZ	HB
1	0.3% Ni/CdS	Ar	DOB					
2	0.3% Ni/CdS	Ar	BZ					
3	0.3% Ni/CdS	Ar	BH					
4	0.3% Ni/CdS	air	BA	74%	96.5%	1.04%	0.6%	0.2%
5	0.3% Ni/CdS	O_2_	BA	46.8%	83.1%	2.6%	12.9%	2.5%
6	0.3% Ni/CdS	Ar	BA	100%	15%	1.2%	81.5%	3.2%
7	0.3% Ni/CdS	N_2_	BA	100%	16.7%	3.8%	79.8%	1.1%
8	0.3% Ni/CdS	He	BA	100%	16.8%	2.6%	81%	1.5%
9	0.3% NiS_2_/CdS	Ar	BA	100%	13.6%	34.5%	34.6%	14.9%
10	0.3% Ni/CdS(E)	Ar	BA	100%	12.3%	5.5%	72.4%	7.8%

a Typical reaction conditions: the reactant is 10 mg; the photocatalyst is 10 mg; CH_3_CN is 5 mL; Ar is at 1 atm; the visible light power is 0.35 W cm^–2^; 5 h.

Before evaluating the photocatalytic performance of CdS and Ni/CdS photocatalysts developed in this study, it is essential to optimize the atmosphere in the reaction system to achieve the high selectivity of desirable products. As shown in entries 4 and 5 in Table 1, 74 and 46.8% of BA are converted to 96.5 and 83.1% of BH as an oxidized byproduct under air and oxygen atmospheres, respectively. The results indicate a negative effect of oxygen on the generation of DOB and BZ. The photocatalytic results in the inert atmospheres (N_2_, He, and Ar) are presented in entries 6–8 in Table 1, which show a similar conversion rate of BA and selectivity to desirable products. In detail, BA completely converts to around 81% BZ and around 3% DOB using 0.3% Ni/CdS photocatalysts under different inert atmospheres after 5 h of visible light irradiation. Hence, the following experiments are performed in an Ar inert atmosphere.

The preparation of Ni/CdS photocatalysts through an in situ photodeposition process is an effective method to improve the conversion rate of BA to BZ with high selectivity. Under visible light irradiation, CdS generates electrons to reduce NiCl_2_ precursors to metallic Ni and then Ni is adsorbed on the CdS surfaces, forming Ni cluster-decorated CdS photocatalysts.^33^ Ni clusters have been considered as the active sites to improve the photocatalytic performances in various applications, such as hydrogen evolution^31,33^ and coupling of amines to imines.^29,34^ To further validate the importance of Ni clusters on CdS surfaces for conversion of BA to BZ, NiS_2_/CdS photocatalysts prepared by using an in situ hydrothermal method and Ni/CdS(E) photocatalysts prepared by using a traditional photodeposition method were compared (both synthesizing details are provided in the Supporting Information). As shown in entry 9 in Table 1, only 34.6% of BZ is generated after 5 h of visible light irradiation, indicating that NiS_2_ on the CdS surfaces cannot significantly improve the conversion rate of BA to BZ. In addition, 0.3% Ni/CdS(E) photocatalysts generate 72.4% of BZ after 5 h of irradiation (entry 10 in Table 1), and 0.3% Ni/CdS provides 81.5% selectivity of BZ. The results demonstrate that Ni clusters on the CdS surfaces significantly improve the yield of BZ. Furthermore, the better photocatalytic performance using 0.3% Ni/CdS than 0.3% Ni/CdS(E) might be attributed to the optimization of the distribution and size of Ni clusters on the CdS surfaces in the presence of BA during the in situ photodeposition process, which is more conducive to the conversion of BA to BZ.

**Figure 2:**
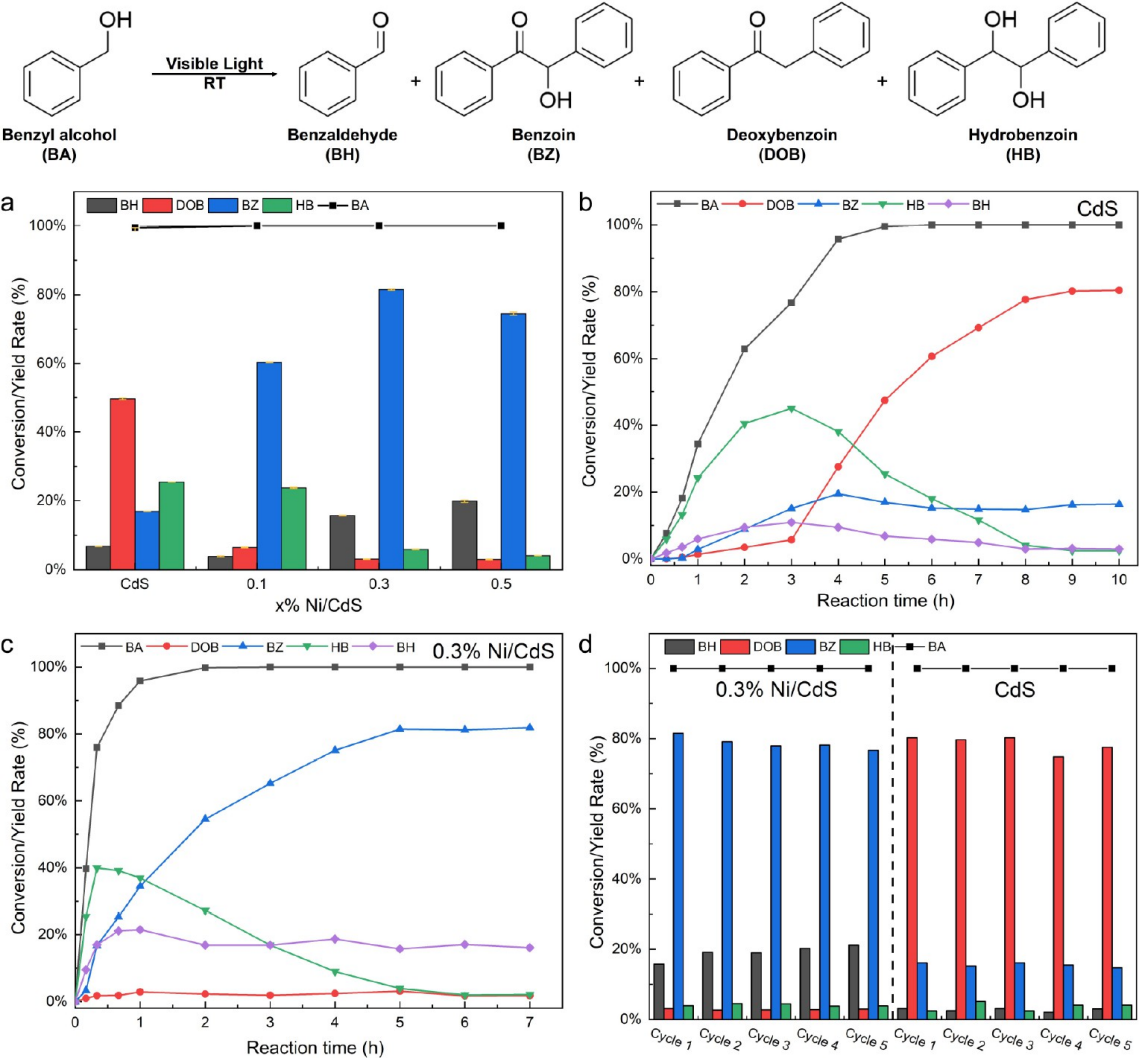
(a) Photocatalytic performance in C–C coupling of BA to corresponding products with CdS and *x*% Ni/CdS after 5 h of visible light irradiation. Reaction kinetics of photocatalytic C–C coupling of BA was studied using (b) CdS and (c) 0.3% Ni/CdS. (d) Stability test of the CdS and Ni/CdS photocatalysts. Reaction conditions: BA is 10 mg; the photocatalyst is 10 mg; CH_3_CN is 5 mL; Ar is at 1 atm; the visible light power is 0.35 W cm^–2^.

The CdS and *x*% Ni/CdS photocatalysts show promising potential in C–C coupling of BA with high selectivities of DOB and BZ, respectively. As shown in Figure 2a, CdS photocatalysts achieve complete conversion of BA to 50% of DOB and 17% of BZ as desirable products after 5 h of visible light irradiation. In this reaction, 25.4% of HB intermediates remain, indicating that the reaction time needs to be extended to achieve their complete conversion. The results demonstrate that CdS nanoparticles have a high potential in conversion of BA to high productivity of DOB. All *x*% Ni/CdS photocatalysts also show complete conversion of BA to HB intermediates in the first reaction step after 5 h of visible light irradiation. In the second reaction step, *x*% Ni/CdS shows significantly improved capability in the conversion of HB intermediates. The residual HB intermediates reduce from 23.8% in 0.1% Ni/CdS to only 4.1% in 0.5% Ni/CdS, indicating that the conversion rate of HB intermediates increases with Ni content on CdS photocatalysts. The selectivity of BZ as the desirable product increases from 60.4% in 0.1% Ni/CdS to 81.5% in 0.3% Ni/CdS but decreases to 74.5% in 0.5% Ni/CdS. The 0.3% Ni/CdS photocatalysts show the highest selectivity of BZ compared to other photocatalysts in this work.

### 3.3 C–C Coupling of BA to DOB and BZ through a Two-Step Reaction

The C–C coupling of BA to DOB and BZ contains two reaction steps, which are (1) C–C coupling of BA to HB intermediates and (2) either dehydration of HB to DOB or dehydrogenation of HB to BZ.^13−15,35,36^ The reaction kinetics of CdS and 0.3% Ni/CdS photocatalysts were determined to investigate the two-step reaction in the C–C coupling of BA to DOB and BZ, respectively. As shown in Figure 2b, using CdS photocatalysts, BA can achieve complete conversion to corresponding products after 5 h of visible light irradiation. In the first 3 h, the primary product is HB intermediates, with a yield of 45%. Afterward, the yield of HB gradually decreases to 2.4%, and the generation of DOB significantly increases from only 5.7% after 3 h to 80.4% after 9 h. Similarly, as shown in Figure 2c, 0.3% Ni/CdS photocatalysts achieve complete conversion of BA to corresponding products after 2 h of visible light irradiation. The yield of HB intermediates significantly increases to 40% after the first 20 min and then gradually decreases to 1.1% after 5 h. Meanwhile, the yield of BZ as the desirable product significantly increases to 81.5% after 5 h, while the generation of DOB retains a minimal selectivity at 1.2%. Both reaction kinetics confirm that DOB and BZ are generated through a two-step reaction pathway. As a result, CdS photocatalysts can achieve complete conversion of BA to 80.4% of DOB after 9 h of visible light irradiation, while 0.3% Ni/CdS photocatalysts promote a complete conversion of BA to 81.5% of BZ after only 5 h. For better comparison with previous works, the apparent quantum yield (AQY) for generation of desirable products was calculated based on the method provided in previous studies,^35,36^ and the calculated details are presented in the Supporting Information. In our reaction system, the calculated AQY for BZ generation using 0.3% Ni/CdS photocatalysts is 4.42%, while the calculated AQY for DOB generation using CdS photocatalysts is 3.38%. Compared with previous studies, our results demonstrate the highest AQY for DOB generation using CdS and for BZ generation using 0.3% Ni/CdS (Table S2).^13,14,36,37^

**Scheme 2:**
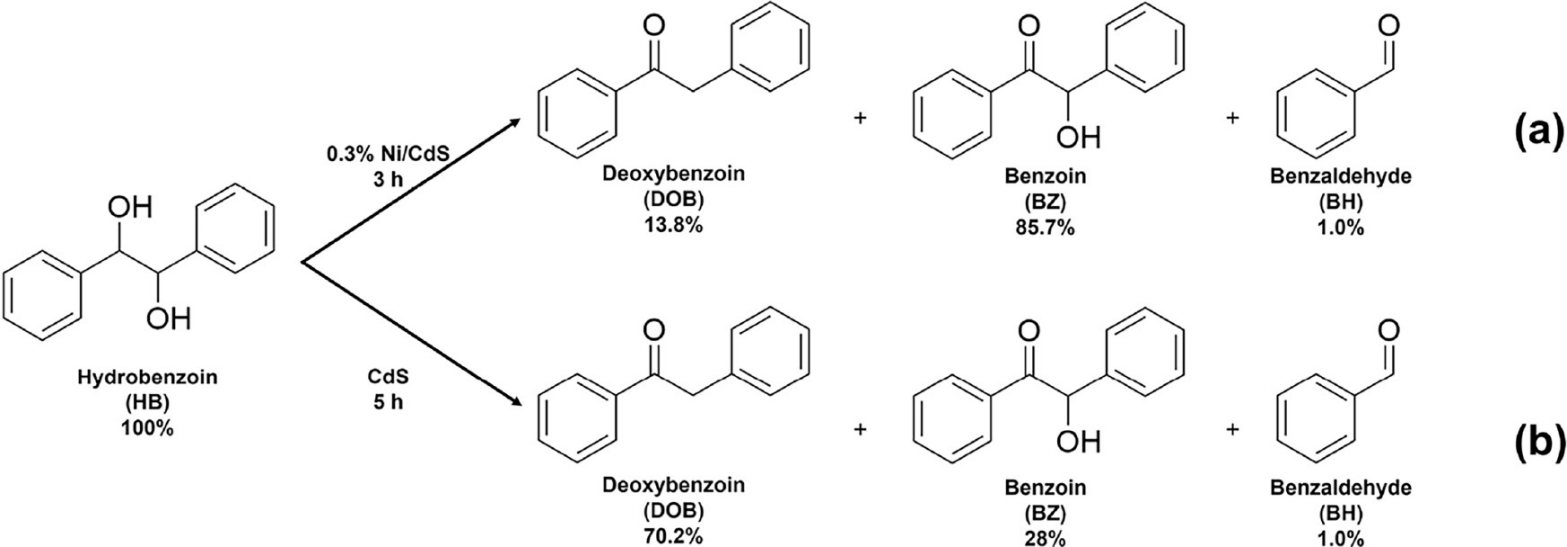
Photocatalytic Conversion of HB Intermediates to DOB, BZ, and BH Using (a) 0.3% Ni/CdS and (b) CdS Reaction conditions: HB is 10 mg; the photocatalyst is 10 mg; CH_3_CN is 5 mL; Ar is at 1 atm; the visible light power is 0.35 W cm^–2^.

To further investigate the two-step reaction in C–C coupling of BA into DOB and BZ, HB intermediates as the reactant were used to generate DOB and BZ by using CdS and Ni/CdS photocatalysts, respectively. As shown in Figure S8, in C–C coupling of the BA reaction, GC-MS spectra reveal two different peaks of HB intermediates, corresponding to different chiral forms of HB, which might be (R,R)-HB, (S,S)-HB and meso-HB. To evaluate the potential impact of these chiral forms on the conversion rate to DOB and BZ, (R,R)-HB, (S,S)-HB, and meso-HB were separately used as reactants to generate BZ and DOB. As shown in Table S3, the selectivity of BZ from three chiral forms of HB shows similar results that are around 86% when using 0.3% Ni/CdS after 3 h of visible light irradiation. The results indicate that the chiral forms of HB do not influence its conversion rate to DOB or BZ. Hence, meso-HB was used as the reactant for the following experiments. As shown in Scheme 2, 0.3% Ni/CdS photocatalysts can achieve complete conversion of HB to generate 85.7% BZ and 13.8% DOB after 3 h of visible light irradiation. Also, CdS photocatalysts can completely convert HB to 70.2% of DOB and 28% of BZ after 5 h. The selectivity of DOB from the conversion of HB using CdS is similar to that from C–C coupling of BA using the same photocatalyst, and the selectivity of BZ from the conversion of HB using 0.3% Ni/CdS is also similar to that from the C–C coupling of BA using the same Ni/CdS catalyst. These results confirm that the selectivity of DOB and BZ is primarily determined by the conversion of HB intermediates in the second reaction step.

### 3.4 Photostability and Feasibility of CdS and Ni/CdS Photocatalysts

**Scheme 3:**
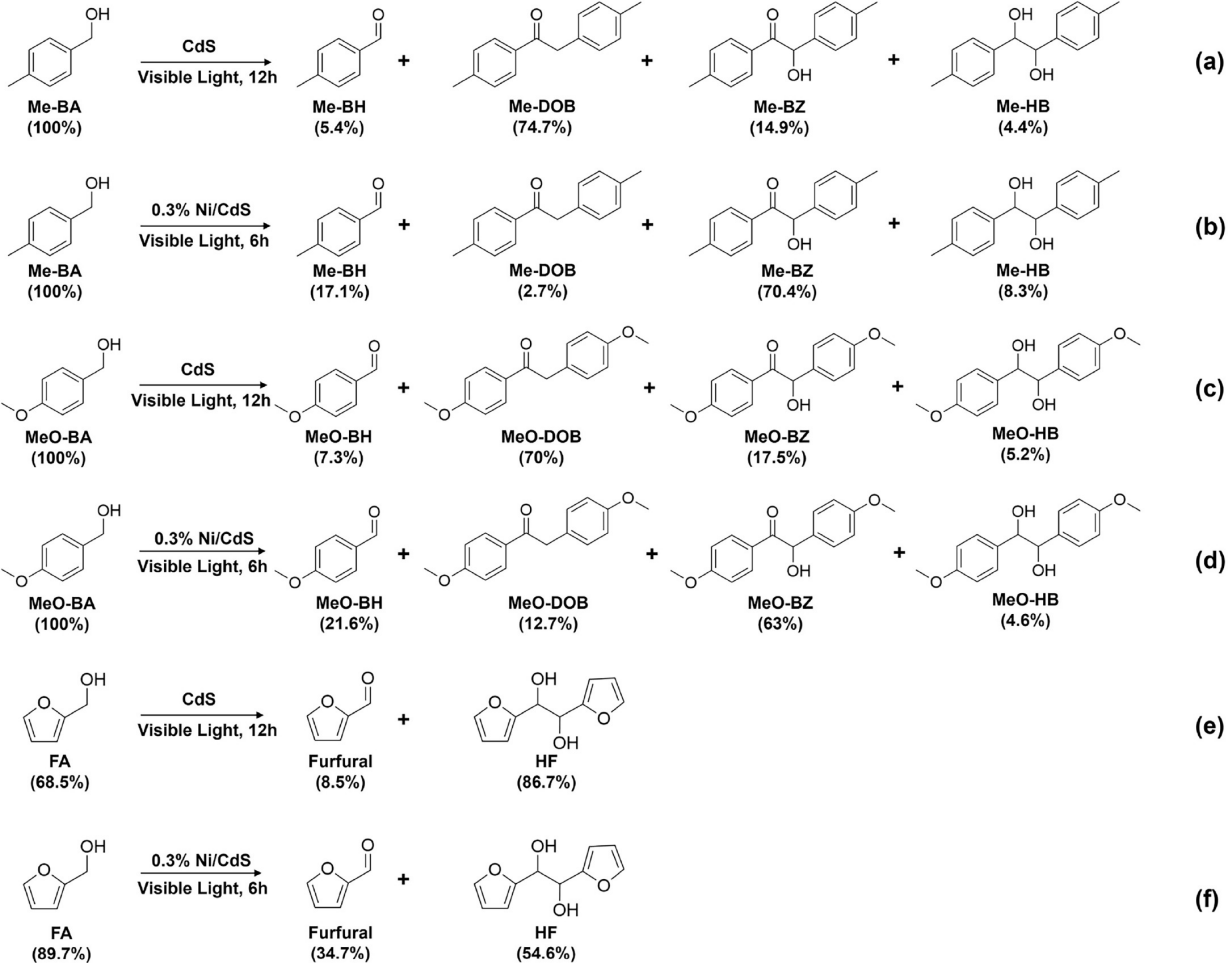
Photocatalytic Conversion of Different Alcoholic Compounds to Corresponding C–C Coupled Products Using CdS and 0.3% Ni/CdS Photocatalysts (a, b) Me-BA; (c, d) MeO-BA; (e, f) FA. Reaction conditions: the reactant is 10 mg; the photocatalyst is 10 mg; CH_3_CN is 5 mL; Ar is at 1 atm; the visible light power is 0.35 W cm^–2^.

The feasibility and photostability of CdS and Ni/CdS photocatalysts are important factors in evaluating the long-term application of photocatalysts in the C–C coupling of alcoholic compounds to desirable products. In the fragmentation of biomass to alcohol-type derivatives, the typical derivatives from lignin conversion are BA with substitutes of −Me and −OMe at the para-position (Me-BA and MeO-BA), as both substitutes are major groups in the native lignin structures.^14^ Additionally, FA is a basic alcohol-type unit in cellulose and hemicellulose.^38−43^ To evaluate the feasibility of CdS and Ni/CdS photocatalysts, Me-BA, MeO-BA, and FA were used to investigate their conversion efficiency to the corresponding C–C coupled products. As shown in Scheme 3b,d, 0.3% Ni/CdS photocatalysts can achieve complete conversion of Me-BA and MeO-BA compounds to 70.4% of Me-BZ and 63% of MeO-BZ, respectively, as desirable products after 6 h of visible light irradiation. In addition, using CdS photocatalysts, the conversion rate of both Me-BA and MeO-BA achieves 100%, with the selectivity of the corresponding DOB products at 74.7% of Me-DOB and 70% of MeO–DOB, respectively, after 12 h of visible light irradiation (Scheme 3a,c). The generated products are important compounds in chemical industrial applications. For example, the generated Me-BZ and MeO-BZ are valuable precursors in the synthesis of 5H-1,4-benzodiazepine derivatives, which are important building blocks in various fields, such as organic chemistry, natural product synthesis, biochemistry, and pharmaceutical synthesis.^44,45^ Similarly, the produced Me-DOB and MeO-DOB are also important materials in generation of the diepoxide of bishydroxydeoxybenzoin (BEDB) that is a cross-linker with low flammability for epoxy adhesive chemistry^46^ and in generation of benzothieno[2,3-*b*]quinolones that shows practical applications in synthesis of anticancer agents, antibacterial and antiviral agents, and fluorescent probes and dyes.^47^

We also investigated the C–C coupling of FA to generate the corresponding products. As shown in Scheme 3e,f, FA converts to HF as the sole C–C coupled product, with selectivities of 86.7% when using CdS photocatalysts after 12 h of visible light irradiation and 54.6% using 0.3% Ni/CdS photocatalysts after 6 h. However, HF cannot further convert to dehydrated or dehydrogenated products, which is consistent with many studies that have demonstrated HF as the sole C–C coupled product.^24,48−52^ To investigate the possibility of conversion of HF to dehydrated or dehydrogenated products in our reaction system, the reaction time for FA conversion using both photocatalysts was prolonged to 24 h of visible light irradiation. As shown in Table S4, 100% of FA was converted to 88.6% of HF using CdS photocatalysts, and 100% of FA was converted to 63.4% of HF using 0.3% Ni/CdS. Both results confirm that HF is the sole C–C coupled product and cannot be further converted to dehydrated or dehydrogenated products. Two possible reasons could explain the results. First, the furan groups in HF show a weaker conjugation effect compared to phenyl groups in HB, Me-HB, or MeO-HB,^53,54^ which reduce the efficiency of photogenerated electron migration from photocatalysts to HF.^55−57^ Second, the O atom in furan groups is the electron-withdrawing group (EWG),^58^ decreasing the electron density of O–H or C_α_–H groups in HF.^59−61^ Despite this, HF is a high-value product, as it can be used as the jet fuel precursor to generate C_10_ alkanes for aviation fuel.^24,48−52^ These experiments demonstrate that both CdS and Ni/CdS photocatalysts have promising potential in the conversion of alcoholic compounds into their corresponding C–C coupled products, affirming their high potential feasibility in practical applications.

Photocorrosion and photostability are important factors to investigate the applicability of CdS and Ni/CdS photocatalysts in the photocatalytic C–C coupling of BA into DOB and BZ, respectively. The photocorrosion and photostability of both photocatalysts were evaluated through recycling experiments. As shown in Figure 2d, after 5 recycling tests, the recycled 0.3% Ni/CdS photocatalysts can completely convert BA to around 78% of BZ after 5 h of visible light irradiation. Similarly, the recycled CdS photocatalysts also keep good stability through 5 recycling tests, achieving complete conversion of BA to around 77% of DOB after 9 h. XRD analysis was conducted to evaluate the structural integrity of both CdS and 0.3% Ni/CdS photocatalysts after 5 recycling tests. As shown in Figure S9, both recycled photocatalysts show similar XRD patterns with fresh samples. These results indicate that CdS and 0.3% Ni/CdS photocatalysts have low photocorrosion and high photostability in C–C coupling of BA into desirable products.

### 3.5 Improved Interaction between BA and Photogenerated Charges on 0.3% Ni/CdS to Enhance the Conversion Rate of BA

**Figure 3:**
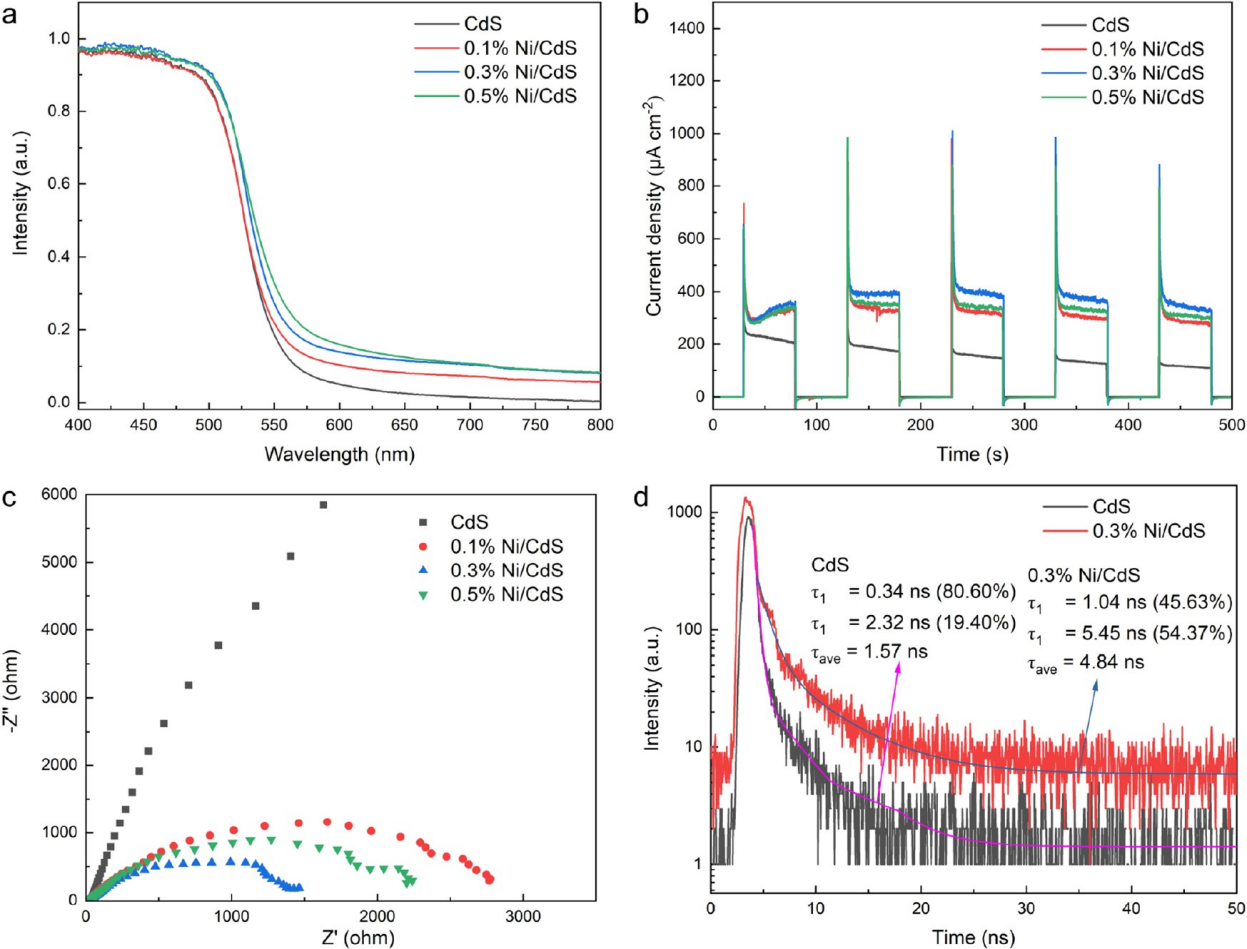
(a) UV–vis diffuse reflectance spectra of CdS and *x*% Ni/CdS photocatalysts. (b) Transient photocurrent of CdS and *x*% Ni/CdS photocatalysts upon turning on and off visible light. (c) EIS results of CdS and *x*% Ni/CdS photocatalysts under visible light irradiation. (d) TRPL spectra of CdS and 0.3% Ni/CdS photocatalysts.

The reaction kinetics in Figure 2b,c show that the conversion rate of BA to BZ using 0.3% Ni/CdS photocatalysts is faster than the conversion rate of BA to DOB using CdS photocatalysts. This improvement is mainly attributed to the fact that the 0.3% Ni/CdS photocatalysts can enhance the visible light absorption capability and improve the photogenerated charge carrier migration efficiency to effectively enhance the interaction between BA and photogenerated charge carriers, therefore increasing the conversion rate in the BA C–C coupling reaction.^15^ Diffuse reflectance spectroscopy (DRS), photoelectrochemical (PEC) measurements, photoluminescence (PL), and time-resolved photoluminescence (TRPL) were employed to examine photophysical properties of CdS and *x*% Ni/CdS photocatalysts. DRS was employed to investigate the visible light absorption capabilities of photocatalysts. As shown in Figure 3a, the red shifts are observed from CdS to 0.5% Ni/CdS photocatalysts, indicating that the visible light absorption capability is increased with the amount of Ni on CdS photocatalysts. The bandgap energy (*E*_g_) of CdS can be determined based on the DRS results, with details provided in the Supporting Information. As shown in Figure S10, the *E*_g_ of CdS is determined to be 2.30 eV. In addition, the *E*_g_ of *x*% Ni/CdS is equal to the *E*_g_ of CdS, as the introduction of Ni clusters on the surfaces of CdS cannot alter the crystal structure of CdS (XRD patterns in Figure S3).^29,31^ Although the introduction of Ni clusters cannot change the *E*_g_ of CdS, it does improve the visible light absorption capability, therefore enhancing the photocatalytic conversion rate of BA to BZ using 0.3% Ni/CdS photocatalysts.

The PEC measurements, which include transient photocurrent responses and electrochemical impedance spectroscopy (EIS), were conducted to investigate the photogenerated charge carrier separation efficiency of CdS and *x*% Ni/CdS photocatalysts. As shown in Figure 3b,c, 0.3% Ni/CdS photocatalysts show the highest photocurrent density and the smallest arc radius compared to other photocatalysts, indicating that 0.3% Ni/CdS photocatalysts significantly enhance the separation efficiency of photoexcited charge carriers and decrease the transfer resistance of charge carriers, therefore enhancing the photocatalytic performance.

PL and TRPL spectra were measured to investigate photogenerated charge carriers’ recombination and migration dynamics. PL emission spectra of CdS and 0.3% Ni/CdS photocatalysts were measured at 400 nm excitation wavelength to investigate the charge carriers’ quenched efficiency. As shown in Figure S11, both photocatalysts exhibit two emission peaks, which are a band-edge emission peak at 559 nm and a defect-mediated emission peak at 595 nm. Generally, the band-edge emission represents the direct recombination of excitons in the photocatalysts, while the defect-mediated emission arises from radiative recombination through defect states.^62,63^ The PL spectrum of phase junction CdS (Figure S11) exhibits that the intensity of defect-mediated emission is significantly higher than that of band-edge emission, indicating that the radiative recombination through defect states is the major pathway for photogenerated charge carriers’ recombination. For the phase junction CdS, the defect-mediated emission might attribute to the formation of defect states by lattice mismatch or interfacial inhomogeneity in the phase junction region.^64−66^ The introduction of Ni clusters on the surfaces of CdS can reduce the intensities of both emissions, as photogenerated electrons transfer from CdS to Ni cocatalysts, which not only facilitates electron separation but also passivates defect states in the phase junction region of CdS photocatalysts.^29,31^ Ni/CdS (0.3%) with lower emission intensities shows enhanced separation efficiency of photogenerated charge carriers and prolonged recombination time, thereby improving the conversion rate of BA to the corresponding products. TRPL was further conducted to investigate their charge carrier decay lifetime. As shown in Figure 3d, the decay curves of two photocatalysts are fitted by two-exponential functions, indicating the presence of two main carrier recombination pathways, corresponding to band-edge recombination (fast decay, τ_1_) and defect-mediated recombination (slow decay, τ_2_).^67−69^ In detail, CdS exhibits τ_1_ and τ_2_ of 0.34 and 2.32 ns, respectively, while 0.3% Ni/CdS shows τ_1_ and τ_2_ of 1.04 and 5.45 ns, respectively. Both longer τ_1_ and τ_2_ in 0.3% Ni/CdS indicate that the introduction of Ni clusters in CdS not only can increase the band-edge recombination time but also prolong the defect-mediated recombination time, which is consistent with the PL results observed for CdS and 0.3% Ni/CdS. The average emission lifetime (τ_ave_) was further calculated based on τ_ave_ =(*A*_1_τ_1_^2^ + *A*_2_τ_2_^2^)/(*A*_1_τ_1_ + *A*_2_τ_2_), where τ_1_ and τ_2_ are the fluorescence lifetime and *A*_1_ and *A*_2_ are pre-exponential factors.^70,71^ The τ_ave_ increases from 1.57 ns in CdS to 4.84 ns in 0.3% Ni/CdS. Both PL spectra and TRPL results demonstrate that the 0.3% Ni/CdS photocatalysts can prolong the residence time of photogenerated charge carriers, as the introduction of Ni clusters on the surfaces of CdS photocatalysts can mainly facilitate the migration of photogenerated electrons from CdS to Ni clusters and passivates defect states in the phase junction region of CdS photocatalysts. In summary, 0.3% Ni/CdS photocatalysts with the highest visible light absorption capability, the highest photoexcited charge carriers’ separation efficiency, the lowest charge carriers’ transfer resistance, and the longest recombination time of charge carriers compared to other photocatalysts can significantly improve the generation of charge carriers and promote the contact between charge carriers and the reactant, therefore improving the conversion rate of BA to BZ.

### 3.6 Mechanistic Study

As discussed in Section 3.3, the C–C coupling of BA into DOB and BZ contains two reaction steps, which are (1) C–C coupling of BA to HB as an intermediate product and (2) dehydration or dehydrogenation of HB to DOB or BZ, respectively. Herein, we delve into the distinct roles played by photogenerated charge carriers and generated radical intermediates in these two reaction steps, aiming to understand the mechanisms in the high selectivity of DOB and BZ using CdS and Ni/CdS photocatalysts, respectively.

#### 3.6.1 C–C Coupling of BA to HB Intermediates through C_α_–H Bond Activation

**Figure 4:**
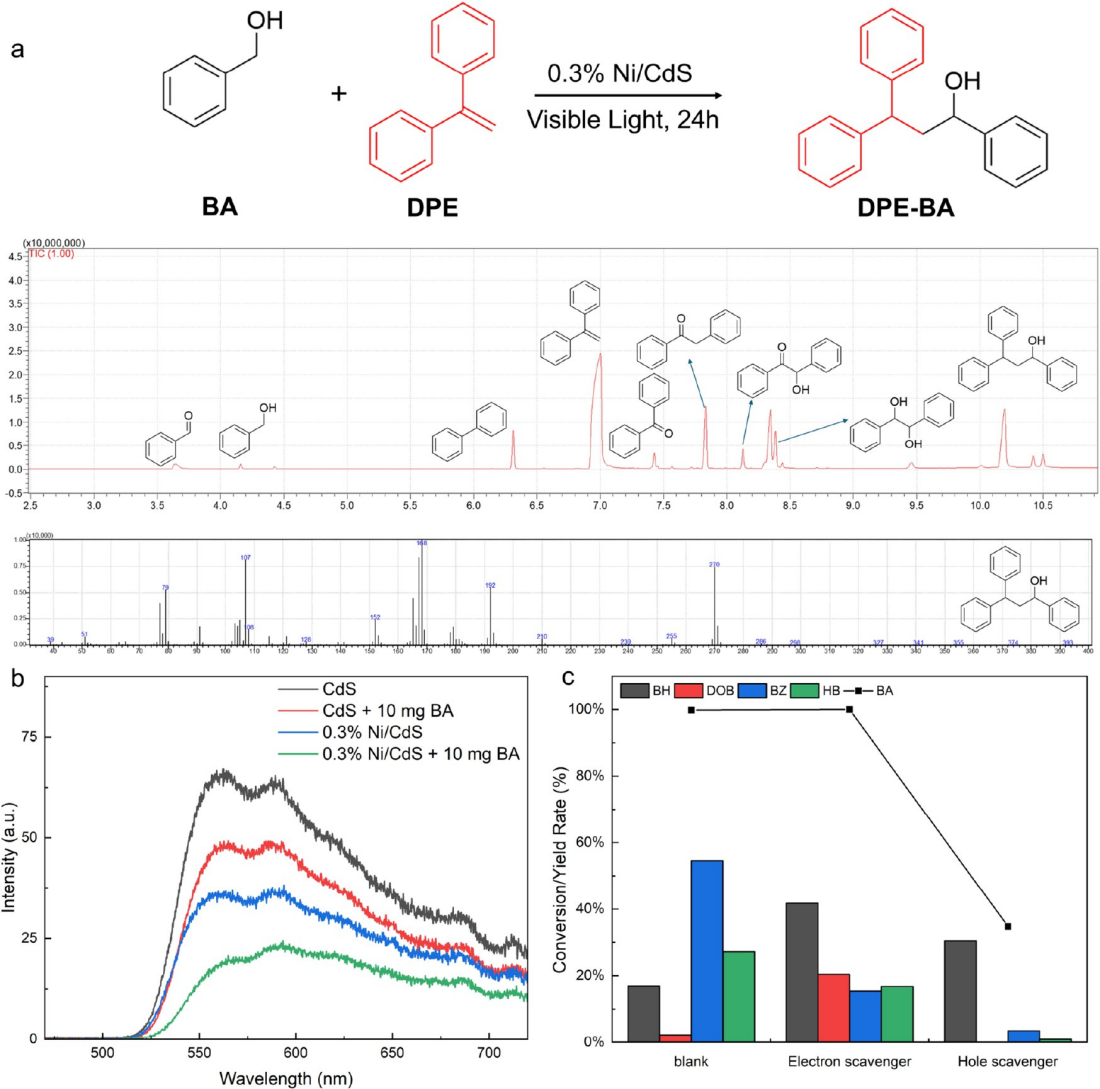
(a) GC-MS spectra of DPE capturing the BA C-radical intermediate and the MS spectrum of DPE-BA. Reaction conditions: BA is 20 mg; DPE is 30 mg; 0.3% Ni/CdS is 20 mg; CH_3_CN is 10 mL; Ar is at 1 atm; the visible light power is 0.35 W cm^–2^; 24 h. (b) PL emission spectra of CdS and 0.3% Ni/CdS with and without 10 mg of BA under visible light irradiation (excitation wavelength at 420 nm). (c) Conversion of BA to corresponding products using different scavengers. Reaction conditions: BA is 10 mg; the photocatalyst is 10 mg; CH_3_CN is 5 mL; Ar is at 1 atm; the visible light power is 0.35 W cm^–2^; 2 h. Hole scavengers are 20 mg of Na_2_S and 10 mg of Na_2_SO_3_; electron scavengers are 30 mg of Na_2_S_2_O_8_.

The C–C coupling of BA to HB intermediates is the first step in the generation of DOB and BZ as desirable products. Previous studies demonstrated that the C_α_–H bond activation in BA is a crucial step for C–C coupling of BA to HB.^13−15,72^ In this study, DPE as the radical trapping agent was used to capture the BA C-centered radical intermediates after C_α_–H bond activation. As shown in Figure 4a, DPE is added into the reaction system, which can significantly suppress the conversion rate of BA and the selectivity of C–C coupled products. In detail, 90% of BA converts to only 2.2% of DOB, 34.3% of BZ, and 15.8% of HB intermediates as the C–C coupled products after 24 h of visible light irradiation. The inability to achieve stoichiometric balance in this result is attributed to the fact that the BA C-centered radical intermediates are captured by DPE to generate DPE-BA (Figure 4a), instead of generation of C–C coupled products. These results demonstrate that C–C coupling of BA involves the radical intermediate mechanism, and the C_α_–H bond activation in BA can determine the generation of HB intermediates.

In the first reaction step, both photogenerated holes and electrons play important roles in the C–C coupling of the BA into HB intermediates. PL analysis was conducted to confirm the charge transfer event between photogenerated charge carriers and BA, as the fast transfer efficiency facilitates the generation of HB intermediates. As shown in Figure 4b, the emission intensities of excited states of both CdS and 0.3% Ni/CdS photocatalysts are relatively low in the presence of 10 mg of BA compared to that in the absence of BA under an excitation wavelength of 420 nm, indicating that the photogenerated charges are quenched with BA. The results demonstrate that the photogenerated holes and electrons transfer from photocatalysts to BA, therefore prolonging the recombination time of charge carriers and improving the efficiency of charge carriers’ utilization. In addition, the emission intensities of 0.3% Ni/CdS in both the presence and absence of 10 mg of BA are lower than those of CdS. The results further confirm that the efficiency of photogenerated charge carriers’ utilization in 0.3% Ni/CdS is higher than in CdS, resulting in a faster conversion rate of BA using 0.3% Ni/CdS compared to using CdS (Figure 2b,c). To investigate their individual roles, hole scavengers and electron scavengers were separately employed to hinder the photogenerated holes and electrons on photocatalysts, respectively. As shown in Figure 4c, the addition of electron scavengers (30 mg of Na_2_S_2_O_8_) in the reaction system shows that BA can still achieve complete conversion, but the yield of the BH byproduct significantly increases from 16 to 41.7% using 0.3% Ni/CdS after 2 h of visible light irradiation, demonstrating that the photoexcited electrons (e^–^) mainly improve the C–C coupling of BA to HB intermediates and decrease the selectivity of the BH byproduct. The addition of hole scavengers (20 mg of Na_2_S and 10 mg of Na_2_SO_3_) significantly decreases the conversion rate of BA from 100 to 34.7% and dramatically increases the yield of the BH byproduct from 16 to 88% after 2 h of visible light irradiation, indicating that the C_α_–H bond activation in BA is mainly determined by the photogenerated holes (h^+^). These trapping experiments show that both h^+^ and e^–^ can collaboratively improve the C_α_–H bond activation in BA and enhance the conversion rate of BA into HB intermediates.

#### 3.6.2 Conversion of HB Intermediates into DOB through C_α_–H Bond Activation or into BZ through O–H Bond Activation

As discussed in Section 3.2, the selectivity of DOB or BZ from C–C coupling of BA is mainly determined by the second reaction step. In detail, HB intermediates can be dehydrated by CdS photocatalysts to improve the selectivity of DOB or dehydrogenated by 0.3% Ni/CdS photocatalysts to enhance the selectivity of BZ. Herein, the core question about the photocatalytic C–C coupled mechanism is how do CdS and 0.3% Ni/CdS photocatalysts exhibit high selectivity for the conversion of HB intermediates to DOB and BZ, respectively.

**Figure 5:**
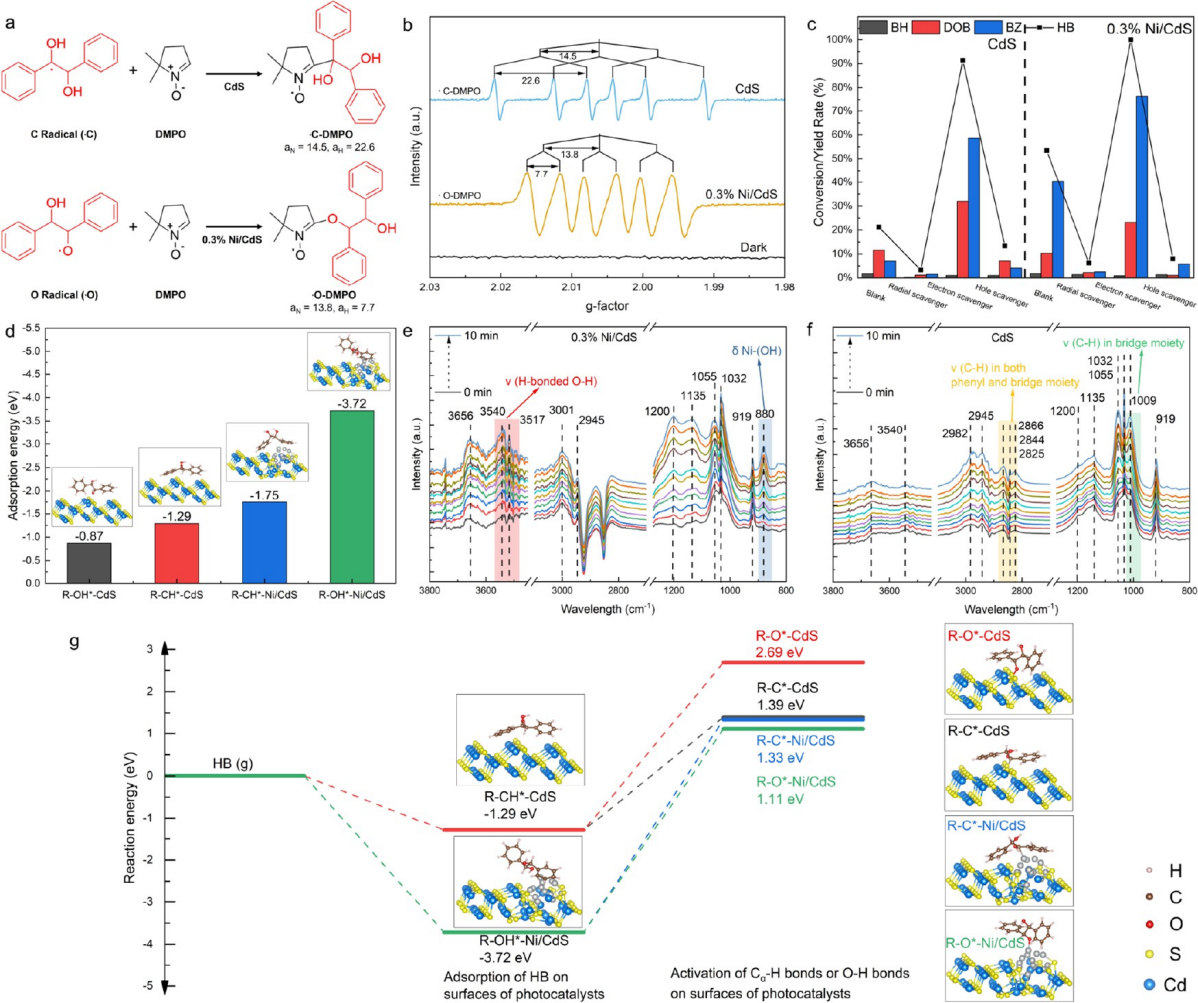
(a, b) In situ ESR spectra in conversion of HB intermediates to DOB and BZ using CdS and 0.3% Ni/CdS photocatalysts after 30 min of visible light irradiation in the presence of DMPO as a spin-trapping agent. The details are provided in the Supporting Information. (c) Conversion of HB intermediates to corresponding products with different scavengers using CdS and 0.3% Ni/CdS photocatalysts. Reaction conditions: HB is 10 mg; the photocatalyst is 10 mg; CH_3_CN is 5 mL; Ar is at 1 atm; the visible light power is 0.35 W cm^–2^; 20 min. Hole scavengers are 20 mg of Na_2_S and 10 mg of Na_2_SO_3_; electron scavengers are 30 mg of Na_2_S_2_O_8_, and free radical scavengers are 30 mg of DMPO. (d) Calculated adsorption energy of HB on surfaces of CdS and Ni/CdS photocatalysts. (e, f) Time-resolved ATR spectra for the adsorption of HB on 0.3% Ni/CdS and CdS. (g) Calculated reaction energy of C_α_–H bond activation and O–H bond activation in HB intermediates on surfaces of CdS and Ni/CdS photocatalysts.

To understand the mechanism in the generation of DOB and BZ from the conversion of HB intermediates, in situ electron spin resonance (ESR) spectroscopy with DMPO as a spin-trapping agent was conducted to identify the generated radical intermediates from HB using CdS and 0.3% Ni/CdS photocatalysts, respectively. As shown in Figure 5a,b, using CdS photocatalysts, sextet signals with a ratio of 1:1:1:1:1:1 (α_N_ = 14.5; α_H_ = 22.6) are observed after 30 min of visible light irradiation, corresponding to the formation of HB C-centered radical intermediates (•C), whereas using 0.3% Ni/CdS photocatalysts, sextet signals with a ratio of 1:1:1:1:1:1 (α_N_ = 13.8; α_H_ = 7.7) are identified, representing the formation of HB O-centered radical intermediates (•O).^22,24,29,73^ Generally, the HB C-centered radical intermediates are generated through the C_α_–H bond activation in HB, whereas the HB O-centered radical intermediates are generated through the O–H bond activation in HB.^22^ These results indicate that CdS photocatalysts have a high potential for the activation of C_α_–H bonds in HB intermediates to generate DOB, and 0.3% Ni/CdS photocatalysts show a high potential for the activation of the O–H bonds in HB intermediates to generate BZ.

The adsorption orientations of HB intermediates on both photocatalysts are the key factors to promote C_α_–H bond activation and O–H bond activation. Generally, the adsorption of HB on both photocatalyst surfaces contains two distinct orientations, which are (1) the orientation of O–H groups in HB toward the photocatalyst surfaces (R-OH*) and (2) the orientation of C_α_–H groups in HB toward the photocatalyst surfaces (R-CH*).^73^ Hence, DFT calculations and time-resolved ATR analyses were conducted to investigate the adsorption orientations of the HB intermediates on both photocatalysts. DFT simulations were first used to calculate the adsorption energies of HB intermediates with two adsorption orientations (R-OH* and R-CH*) on CdS and Ni/CdS photocatalysts. We constructed the cubic CdS (220) surfaces as the CdS photocatalyst reaction platform, as our previous work has demonstrated that the cubic (220) facets on phase junction CdS photocatalysts play a crucial role in the C_α_–H bond activation in lignin to generate desirable aromatic monomers.^28^ Ni/CdS was built by loading Ni_13_ clusters onto the created cubic CdS (220) surfaces for HB adsorption and both bond activations (Figure S12). As shown in Figure 5d, on the CdS surfaces, the adsorption energy of R-OH* at −0.87 eV is higher than that of R-CH* at −1.29 eV, indicating a preference for HB adsorption with the R-CH* orientation. Conversely, on the Ni/CdS surfaces, the adsorption energy of R-OH* at −3.72 eV is significantly lower than that of R-CH* at −1.75 eV, suggesting a predilection for HB adsorption with an R-OH* orientation. To further investigate the adsorption orientations of HB intermediates on both photocatalysts, time-resolved ATR analyses were conducted. As shown in Figure 5e,f, several peaks are observed from 3800 to 2800 cm^–1^ and from 1200 to 800 cm^–1^, and their relevant detected bands and assignment are listed in Table S5. For 0.3% Ni/CdS photocatalysts, two peaks of 3540 and 3517 cm^–1^ (red area) are observed and gradually increase with the adsorption time, corresponding to H-bonded −OH groups on photocatalyst surfaces (ν (H-bonded O–H)), and another peak of 880 cm^–1^ (blue area) is also observed, representing the bending vibration of Ni–OH bonds (δ Ni–(OH)).^74^ The results indicate that the adsorption orientation of HB on Ni/CdS photocatalysts is mainly R-OH*. For CdS photocatalysts, four peaks occur in comparison with the ATR spectra of 0.3% Ni/CdS, which are 2866, 2844, 2825, and 1009 cm^–1^. The intensity of these peaks gradually increases with the adsorption time. The peaks between 2866 and 2825 cm^–1^ (orange area) represent the stretching vibration of C–H bonds (ν (C–H)) in both phenyl (C_6_H_5_) and the bridge moiety (CH–CH) of HB intermediates, and a peak of 1009 cm^–1^ (green area) corresponds to ν (C–H) in the bridge moiety (CH–CH) of HB intermediates.^75^ The results confirm that the adsorption orientation of HB on CdS photocatalysts is R-CH*. Based on DFT and in situ ATR results, the C_α_–H groups in HB intermediates tend to orient toward the CdS surfaces (R-CH*) and the O–H groups in HB tend to orient toward the Ni/CdS surfaces (R-OH*).

DFT calculations were further conducted to investigate the reaction energies of C_α_–H bond activation and O–H bond activation in HB intermediates based on adsorption of HB on CdS surfaces with an R-CH* orientation and on Ni/CdS surfaces with an R-OH* orientation. As shown in Figure 5g, on CdS surfaces, the bond dissociation energy (BDE) of O–H bond activation to form R-O* is 2.69 eV, which is significantly higher than the BDE of C_α_–H bond activation at 1.39 eV to generate R-C*. Conversely, on the Ni/CdS photocatalyst surfaces, the BDE of the activation of the O–H bond to form R-O* is 1.11 eV, which is lower than that of the activation of the C_α_–H bond at 1.33 eV to generate R-C*. In summary, CdS photocatalysts show the adsorption of HB with an R-CH* orientation, which mainly activates the C_α_–H bonds in HB intermediates and improves the generation of DOB, while Ni/CdS photocatalysts show the adsorption of HB with an R-OH* orientation, which predominantly activates the O–H bonds in HB and enhances the selectivity of BZ.

The in situ ESR and DFT results indicate that the generation of HB C-centered radical intermediates through the C_α_–H bond activation and the generation of HB O-centered radical intermediates through the activation of the O–H bond are important factors in determining the yields of DOB and BZ, respectively. Therefore, several radical scavengers were applied to further investigate the generation of both radical intermediates in two reaction systems. As shown in Figure 5c, DMPO was added into the photocatalytic system to quench both generated radical intermediates to investigate their effect on the photocatalytic performance in conversion of HB to DOB and BZ. The results show that the conversion rate of HB is significantly suppressed to 3.3% by addition of DMPO in the CdS reaction system and to 6.2% in the 0.3% Ni/CdS reaction system after 20 min of visible light irradiation, indicating that the formation of both radical intermediates is essential to improve the conversion rate of HB to DOB and BZ. DMPO is a general free radical scavenger that can capture both radicals in our reaction systems. To specifically evaluate the generation of HB C-centered radicals or HB O-centered radicals using both photocatalysts, MEOH and DPE were further conducted to hinder the specific radicals and investigate the photocatalytic performances. MEOH is a common scavenger to quench hydroxyl radicals (•OH) and R-O• radicals, as reported in previous studies.^76−78^ In our reaction systems, the main radicals are HB C-centered or HB O-centered (R-O•) radicals. Therefore, the addition of MEOH can capture the generated HB O-centered radicals. As shown in Figure S13, the addition of 30 mg of MEOH improves the conversion rate of HB from 21.2 to 30.5% using CdS and from 53.4 to 90.6% using 0.3% Ni/CdS photocatalysts. Also, the yields of DOB are significantly improved from 11.6 to 22.9% using CdS and from 10.3 to 64.1% using 0.3% Ni/CdS. The results further demonstrate that the generation of BZ is mainly determined by the formation of HB O-centered radicals. Previous studies demonstrated that DPE scavengers could preferentially quench C-centered radicals.^14,79^ In our reaction system, the additions of DPE have the potential to quench the generation of HB C-centered radicals. As shown in Figure S13, the addition of 30 mg of DPE reduces the conversion rate of HB to 8.4% using CdS and 14.4% using 0.3% Ni/CdS. The yields of BZ are higher than that of DOB using both photocatalysts. For CdS, the yields of BZ and DOB are 5.3 and 2.1%, respectively, while for 0.3% Ni/CdS, the yields of BZ and DOB are 10.1 and 3.3%, respectively. The results demonstrate that the generation of BZ in our reaction system is mainly determined by the HB O-centered radicals.

In addition, previous works demonstrated that the selectivity of the DOB and BZ can be determined by the band structure of the photocatalysts. For example, photocatalysts with high reductive capability can enhance the selectivity of DOB by promoting the reductive cleavage of the C_α_–OH bonds in the formed HB C-centered radical intermediates.^14^ Inspired by this study,^14^ the cleavage of C_α_–H bonds in the formed HB O-centered radical intermediates can improve the generation of BZ, and the cleavage of C_α_–H bonds is mainly determined by the photogenerated holes (photo-oxidative capability).^13,14,25,73,80^ Hence, it is important to investigate the band structure of CdS and Ni/CdS photocatalysts, as these photocatalysts exhibit different selectivities in the generation of DOB and BZ. Generally, a lower *E*_CB_ represents a stronger reductive capability, while a higher *E*_VB_ corresponds to a stronger oxidative capability.^81,82^*E*_VB_ was obtained from the XPS-VB results, and *E*_CB_ can be calculated using the equation *E*_CB_ = *E*_VB_ – *E*_g_ (the details are presented in Table S6). As a result, CdS shows *E*_VB_ at 1.17 eV and *E*_CB_ at −1.13 eV, while 0.3% Ni/CdS shows *E*_VB_ at 1.73 eV and *E*_CB_ at −0.57 eV. Therefore, the photo-oxidative capability of 0.3% Ni/CdS is stronger than that of CdS, while the photoreductive capability of CdS is stronger than that of 0.3% Ni/CdS. As a result, 0.3% Ni/CdS with a stronger photo-oxidative capability can improve the cleavage of C_α_–H bonds in the formed HB O-centered radical intermediates to enhance the generation of BZ, while CdS with a stronger photoreductive capability can improve the cleavage of C_α_–OH bonds in the formed HB C-centered radical intermediates to enhance the generation of DOB.

The hole and electron scavengers were further used to hinder the photogenerated holes or electrons in the reaction system and investigate their respective impacts on the conversion of HB intermediates to DOB and BZ. As shown in Figure 5c, the addition of hole scavengers (20 mg of Na_2_S and 10 mg of Na_2_SO_3_) can significantly suppress the conversion rate of HB intermediates on both photocatalysts after 20 min of visible light irradiation, as the first activation step (C_α_–H bond activation or O–H bond activation in HB) requires the photogenerated holes. The addition of electron scavengers (30 mg of Na_2_S_2_O_8_) can significantly improve the reaction rate of HB from 53.4 to 100% using 0.3% Ni/CdS and from 21.2 to 91.3% using CdS photocatalysts after 20 min of visible light irradiation. Also, the yield of BZ dramatically enhances from 40.4 to 76.4% using 0.3% Ni/CdS photocatalysts and from 7 to 58.6% using CdS photocatalysts. The improved conversion rate of HB is mainly attributed to the fact that the addition of electron scavengers can facilitate the consumption of photogenerated electrons and improve the generation of holes, therefore improving the first activation step by holes. The improved yields of BZ on both photocatalysts mainly due to the extra photogenerated holes can facilitate the cleavage of C_α_–H bonds after the first activation step. Based on these analyses, we can conclude that both activations of C_α_–H bonds or O–H bonds in HB require the photo-oxidative capability provided by holes, the cleavage of C_α_–OH bonds in the formed HB C-centered radicals requires stronger photoreductive capability provided by electrons, and the cleavage of C_α_–H bonds in the formed HB O-centered radicals requires stronger photo-oxidative capability.

#### 3.6.3 Photocatalytic Mechanism for C–C Coupling of BA to DOB and BZ

**Scheme 4:**
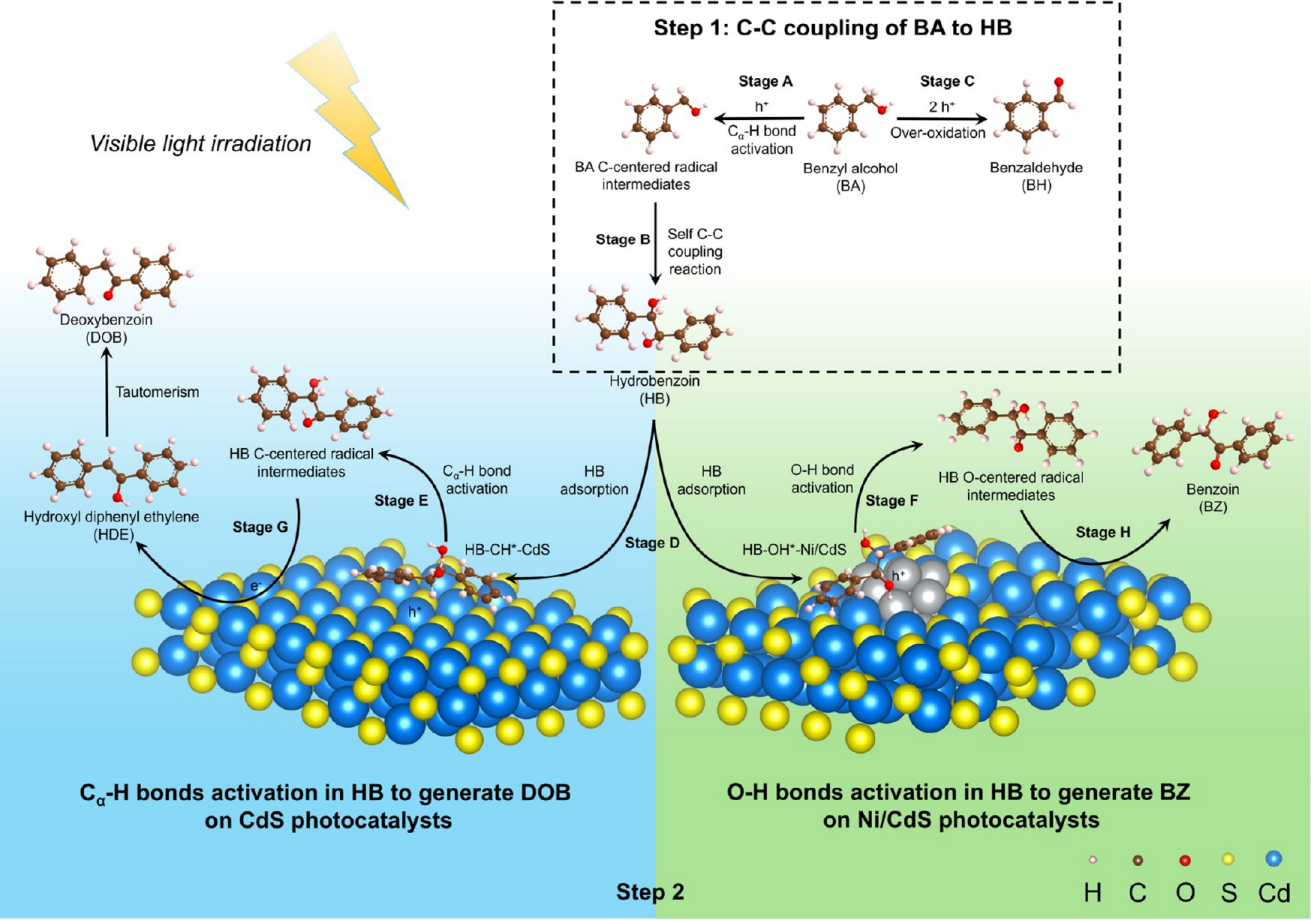
Proposed Mechanism in C–C Coupling of BA to DOB and BZ as Desirable Products through Two-Step Reactions

Based on the results discussed above, we proposed a plausible mechanism for photocatalytic C–C coupling of BA to DOB or BZ with high selectivity as desirable products using CdS or Ni/CdS photocatalysts, respectively. As shown in Scheme 4, the conversion of BA into DOB and BZ requires two reaction steps, which are (1) C–C coupling of BA to HB intermediates and (2) dehydration or dehydrogenation of HB intermediates to DOB or BZ, respectively. In the first reaction step, h^+^ activates the C_α_–H bonds in BA to form BA C-centered radical intermediates and then desorbs on the surfaces of photocatalysts to generate HB as an intermediate product through the self-C–C coupling reaction (Stages A and B). In this step, BH as a byproduct is converted through overoxidation of BA by h^+^ (Stage C). In the second reaction step, HB intermediates adsorb on the photocatalyst surfaces and then activate the C_α_–H bonds to generate HB C-centered radical intermediates on CdS surfaces or activate the O–H bonds to form HB O-centered radical intermediates on Ni/CdS surfaces (Stages D–F). Using the CdS photocatalysts, the C_α_–H groups in HB intermediates preferentially orient toward the photocatalyst surfaces, as the adsorption energy of R-OH* at −0.87 eV is higher than that of R-CH* at −1.29 eV (Figure 5d), therefore improving the preference of C_α_–H bond activation to form HB C-centered radical intermediates (Stage E). In Stage G, the HB C-centered radical intermediates can be reduced by e^–^ to form HDE, which subsequently leads to the generation of DOB through the tautomerization of HDE.^14^ In addition, using Ni/CdS photocatalysts, O–H groups in HB are preferred to orient toward the Ni/CdS surfaces, attributed to the fact that the adsorption energy of R-OH* at −3.72 eV is lower than that of R-CH* at −1.75 eV, therefore improving the activation of the HB O–H bonds to generate HB O-centered radical intermediates (Stage F). In Stage H, the formed O-centered radical intermediates can be further oxidized by h^+^ to generate BZ as a desirable product (Stage H). In summary, the prepared phase junction CdS nanoparticles can significantly improve the generation of DOB by enhancing the C_α_–H bond activation, and the prepared Ni/CdS photocatalysts can enhance the yield of BZ by improving the activation of the O–H bond.

## 4 Conclusions

In this study, we found that the photocatalytic C–C coupling of BA to generate DOB and BZ as desirable products contains two reaction steps, which are (1) C–C coupling of BA to generate HB intermediates and (2) dehydration of HB to DOB or dehydrogenation of HB to BZ. The generated radical intermediates in both reaction steps are important in determining the selectivity of the corresponding products. In detail, in the first reaction step, the activation of C_α_–H bonds in BA through h^+^ forms BA C-centered radical intermediates, and the formed radicals determine the formation of HB intermediates through the self-C–C coupling reaction. In the second reaction step, the selectivity of DOB and BZ is mainly controlled by the activation of C_α_–H bonds to form HB C-centered radical intermediates and by the activation of O–H bonds to form HB O-centered radical intermediates, respectively. The elaborately designed phase junction CdS photocatalysts and Ni/CdS photocatalysts can precisely control the C_α_–H bond activation and the O–H bond activation in HB intermediates by adjusting the adsorption orientation of HB on the photocatalysts surfaces. Using phase junction CdS photocatalysts, the C_α_–H groups in HB tend to orient toward the surfaces (R-CH*), as the adsorption energy of R-CH* at −1.29 eV is lower than that of R-OH* at −0.87 eV. The R-CH* orientation improves the activation of C_α_–H bonds in HB, thereby enhancing the selectivity of DOB. Conversely, using Ni/CdS photocatalysts, the O–H groups in HB prefer to orient toward the surfaces (R-OH*), as the adsorption energy of R-OH* at −3.72 eV is lower than that of R-CH* at −1.75 eV. This orientation enhances the activation of O–H bonds in HB, resulting in an increased selectivity of BZ as the desirable product. As a result, phase junction CdS photocatalysts can achieve complete conversion of BA to 80.4% of DOB after 9 h of visible light irradiation, 0.3% Ni/CdS photocatalysts show the complete conversion of BA to 81.5% of BZ after only 5 h of visible light irradiation, and both elaborately developed photocatalysts exhibit the best photocatalytic performance compared to previous works.

## Supporting Information Available

Experimental details; photocatalytic reaction setup system; HAADF-STEM image; ICP-OES results; XRD patterns; XPS spectra; GC-MS results; GC spectra; ^1^H and ^13^C NMR spectra; photocatalytic performance; band structure; PL spectra (PDF)
