# Supplementary material for: Specific Photocatalytic C–C Coupling of Benzyl Alcohol to Deoxybenzoin or Benzoin by Precise Control of Cα–H Bond Activation or O–H Bond Activation by Adjusting the Adsorption Orientation of Hydrobenzoin Intermediates
Source: ACS Catal. 2024 Oct 2;14(20):15306–24. doi: 10.1021/acscatal.4c03426 (PMC11494511; doi:10.1021/acscatal.4c03426)
Supplement: Supplementary file 1 — cs4c03426_si_001.pdf [file cs4c03426_si_001.pdf]

Supporting Information.

Specific Photocatalytic C-C Coupling of Benzyl Alcohol to Deoxybenzoin or Benzoin by Precise Control of C_{α}-H Bonds Activation or O-H Bonds Activation through Adjusting the Adsorption Orientation of Hydrobenzoin Intermediates

Zongyang Yue [a], Guanchu Lu [a], Wenjing Wei [a], Yanan Deng [a], Luxi Yang [a], Shibo Shao [a,b], Xianfeng Chen [c], Yi Huang [a], Jianhua Qian [d], Xianfeng Fan [a]**

[a] Institute for Materials and Processes, School of Engineering, The University of Edinburgh, Edinburgh, EH9 3BF, U.K.

[b] Petrochemical Research Institute, PetroChina Company Limited, Beijing, 102206, China

[c] Institute for Bioengineering, School of Engineering, The University of Edinburgh, Edinburgh, EH9 3BF, U.K.

[d] School of Petrochemical Engineering, Liaoning Petrochemical University, Fushun, 113001, China

* J. Q. E-mail: qianjianhualn@163.com

* X. F. Tel.: +441316505678; E-mail: x.fan@ed.ac.uk

Determination of Apparent Quantum Yield (AQY)

The AQY for generation of DOB and BZ from C-C coupling of BA is an important factor to determine the photon utilization efficiency. Inspired by previous studies [1,2], a 300 W xenon lamp equipped with a 420 nm UV filter was used to simulate the 420 nm monochromic light illumination. The light power density is measured by a power meter (THORLABS, PM16-144). The following equations were conducted to calculate the AQY:

$$AQY = \frac{K \times moles\ of\ sepcific\ C{-}C\ coupled\ product\ per\ hour}{moles\ of\ photon\ flux\ per\ hour} \times 100\% \quad (1)$$

$$Moles\ of\ photon\ flux\ per\ hour = \frac{I \times A \times t \times \lambda}{N_A \times h \times c} \quad (2)$$

where N_A is Avogadro constant (6.022 × 10^{23} photons mol^{-1}), h is the Planck constant (6.626 × 10^{-34} J s), c is the speed of light (3 × 10^8 m s^{-1}), A is the irradiation area (3.14 cm^2), I is the light power density (0.35 W cm^{-2}), t is time, λ is the incident light wavelength (420 nm). K is number of transferred electrons (in conversion of BA to DOB, K = 5; in conversion of BA to BZ, K = 4).

DFT calculation

DFT calculations were conducted by Vienna ab initio simulation package (VASP5.4.4). The Perdew-Burke-Ernzerhof (PBE) exchange-correlation functional and the projector augmented wave (PAW) method were employed [3–5]. Van der Waals interactions were incorporated using the empirical DFT-D3 method [6]. The Brillouin zone k-points were determined using a Monkhorst-Pack grid mesh with a set of 2×2×1 for all periodic structures, and the cutoff

energy was set at 450 eV. Partial occupancies of the Kohn−Sham orbitals were allowed using the Gaussian smearing method with a width of 0.05 eV. Convergence criteria were established at 0.02 eV $Å^{-1}$ for forces and 10^{-5} eV for energy.

ESR method

The generated radical intermediate was identified using an in-situ electron spin resonance (ESR) spectrometer (Bruker-A300) operating at a frequency of 9.83 GHz. To prepare the samples, 10 mg of BA, 2 mg of photocatalysts, along with 30 μl of 5,5-dimethyl-1-pyrroline-N-oxide (DMPO), were added in 1 mL of dry CH_3CN. The sample was then transferred to the EPR tube, pre-saturated with Ar gas, and subjected to visible light irradiation for 30 min before detection.

Radical Trapping Experiments

To investigate the generation of BA radical intermediates in the photocatalytic C-C coupling of BA to DOB and BZ, DPE was utilized as a trapping agent based on prior studies [7]. Specifically, 30 mg of DPE and 20 mg of BA were dissolved in 10 mL of CH_3CN with 0.3% of $NiCl_2$, and 20 mg of CdS was dispersed. After 30 min of argon purging (10 mL min^{-1}) and 24 h of irradiation by a xenon arc lamp with a 420 nm UV filter (0.35 W cm^{-2}), the solution was processed and analysed by GC-MS.

Preparation of Ni/CdS(E)

The Ni/CdS(E) photocatalysts were synthesized through the traditional photo-deposition method. Specifically, 50 mg of prepared CdS nanoparticles were dispersed in a 5 mL of

ethanol solution containing 0.3% molar ratio of $NiCl_2$ (molar ratio of Ni / 50 mg CdS). The mixed solution was stirred and purged argon in the sealed reactor for 30 min. Then the sealed reactor was illuminated by a xenon arc lamp with a 420 nm UV filter for 1 h. During the irradiation stage, the temperature of solution was kept at 20 °C with cooling water. After the irradiation, the sample was collected by centrifugation (9000 rpm), rinsed several times with ethanol, and then dried under vacuum at 60 °C for 4 h. The obtained solid samples were labelled at 0.3% Ni/CdS(E) and stored under an inert atmosphere condition.

Preparation of NiS_2/CdS

The NiS_2/CdS photocatalysts were synthesized through the in-situ hydrothermal method. Typically, 308 mg of $Cd(NO_3)_2{\cdot}4H_2O$, 0.3% molar ratio of $NiCl_2$ (molar of $NiCl_2$ / molar of 308 mg of $Cd(NO_3)_2{\cdot}4H_2O$), and 150 mg of trisodium citrate were dissolved into 15 mL of water and EG mixed solution (v_{water}/v_{EG} = 1/5). After ultrasonic dispersion for 10 min and then vigorously stirred for 30 min, 375 mg of thioacetamide was added into the solution. After being stirred for another 30 min, the mixture was transferred into a 25 mL stainless Teflon-lined autoclave reactor. The autoclave reactor was subsequently heated to 160 °C with a 3 °C min^{-1} of heating rate in an oven and kept the temperature for 4h. After naturally cool-down, the sample was collected by centrifugation (9000 rpm), rinsed several times with ethanol and water, respectively. The solid samples were then dried under vacuum at 60 °C for 4 h and labelled as 0.3% NiS_2/CdS.

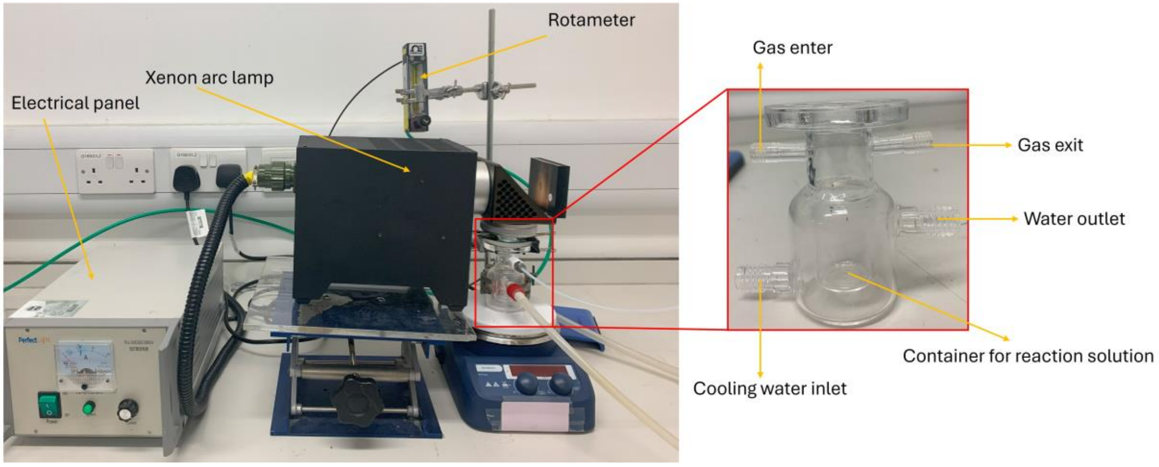

Figure S1. The photocatalytic reaction set-up system.

As shown in **Figure S1**, our reaction system consists of two main parts: the visible light supply and the reaction apparatus. The visible light supply is controlled by an electrical panel to adjust the light intensity, with a Xenon arc lamp equipped with a 420 nm UV filter serving as the visible light source. The reaction apparatus includes a customized quartz reactor for photocatalytic reactions and a rotameter for regulating the flow rate of Ar gas (10 mL min^{-1}). In the reactor setup (right picture), the two bottom ports are designated for the cooling water inlet and outlet, maintaining the reaction solvent at approximately 20 °C. The two upper ports facilitate the entry and exit of Ar gas, ensuring that the reaction system operates under an inert atmosphere.

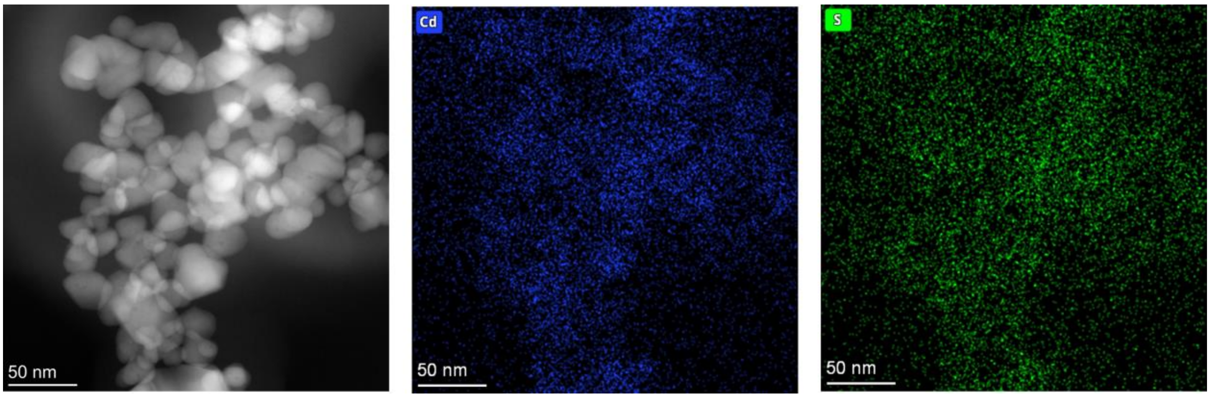

Figure S2. HAADF-STEM image and corresponding elemental mappings of phase junction CdS photocatalysts.

Both HAADF-STEM images of CdS photocatalysts in **Figure S2** and TEM images in **Figure 1a** display comparable irregular nanoparticles with an average particle size of around 30 nm. Furthermore, the corresponding elemental mappings of CdS photocatalysts in **Figure S2** demonstrate that Cd and S elements are uniformly dispersed throughout the particles.

Table S1. The results of elemental analysis by ICP-OES.

	CdS	0.1% Ni/CdS	0.3% Ni/CdS	0.5% Ni/CdS
Ni/Cd	0	0.11%	0.36%	0.43%

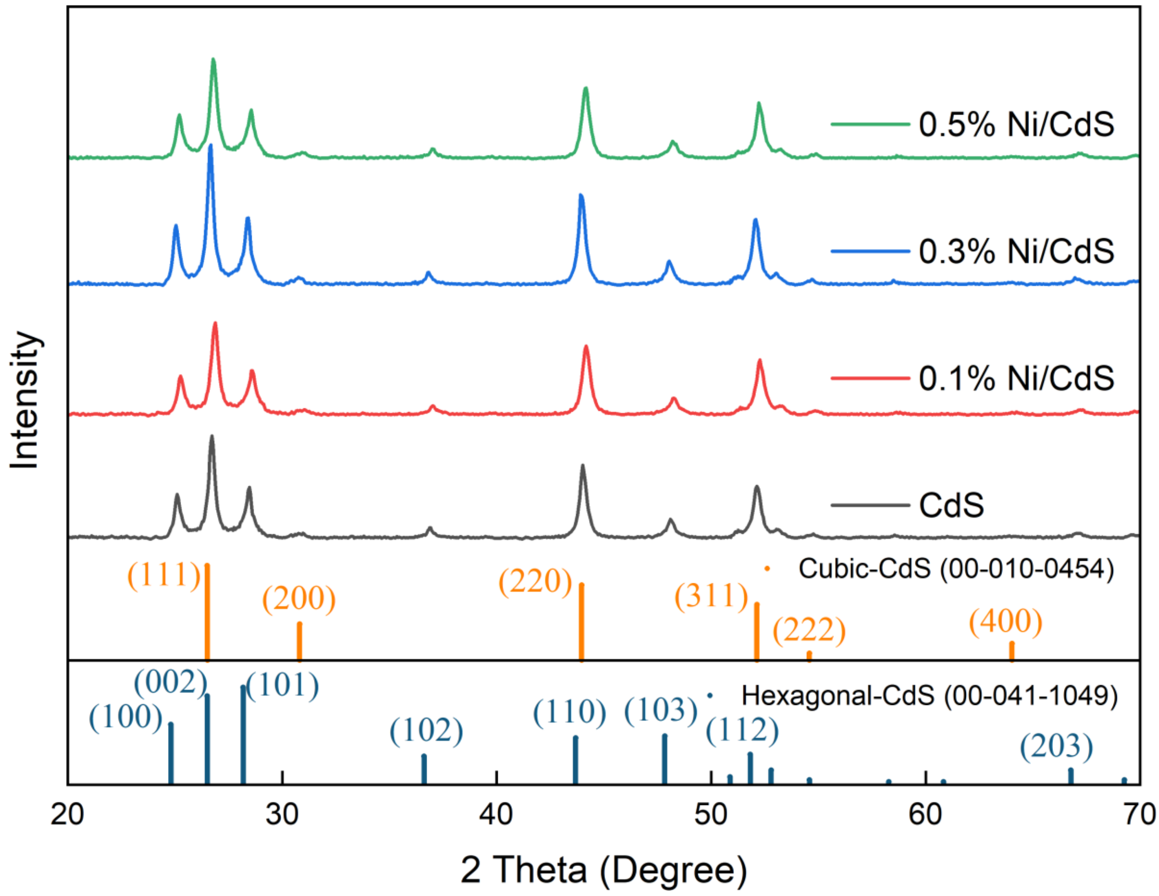

Figure S3. Full XPS spectrum of CdS and 0.3% Ni/CdS photocatalysts. XRD patterns of CdS, 0.1% Ni/CdS, 0.3% Ni/CdS, 0.5% Ni/CdS photocatalysts.

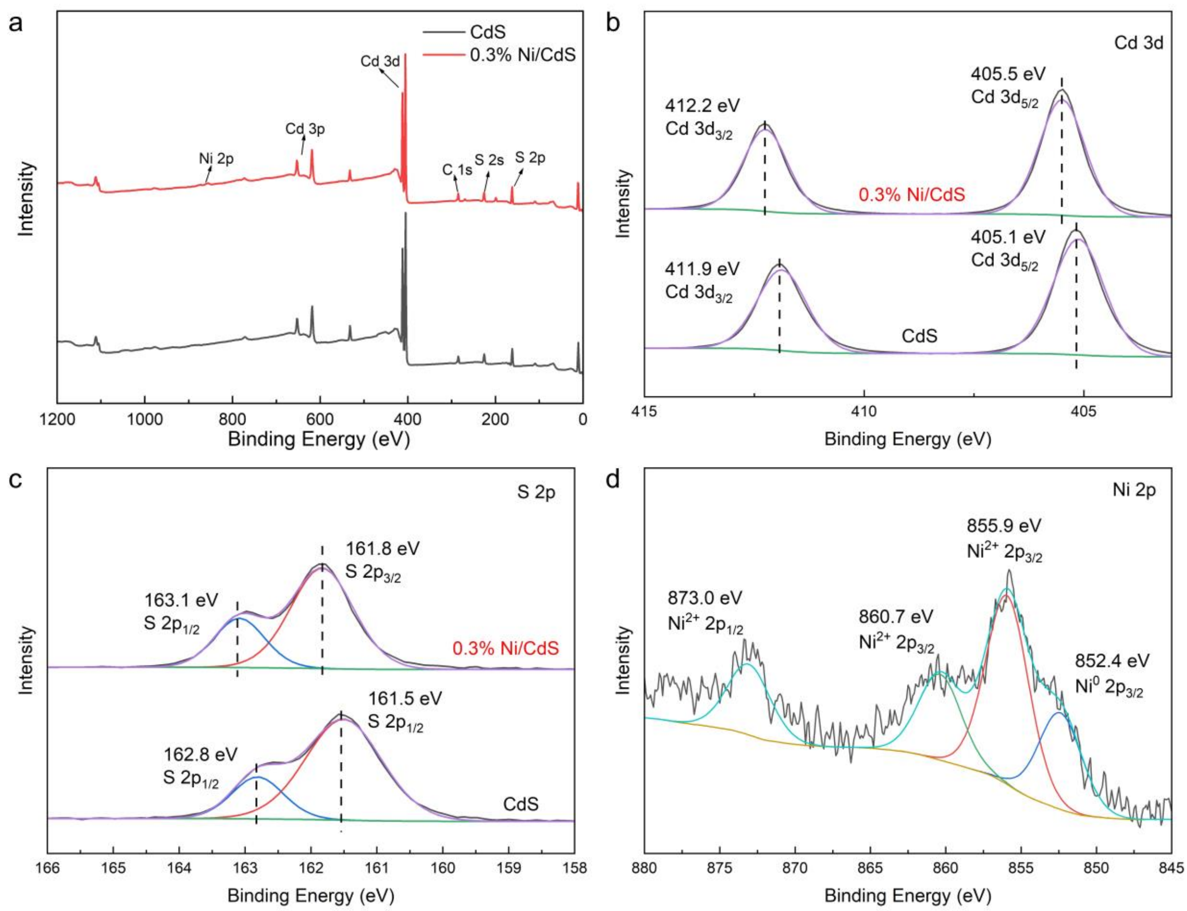

Figure S4. (a) Full XPS spectrum of CdS and 0.3% Ni/CdS photocatalysts. High-resolution XPS spectra of (b) Cd 3d states, (c) S 2p states in phase junction CdS and 0.3% Ni/CdS photocatalysts. (d) High-resolution XPS spectrum of Ni 2p states in 0.3% Ni/CdS photocatalysts.

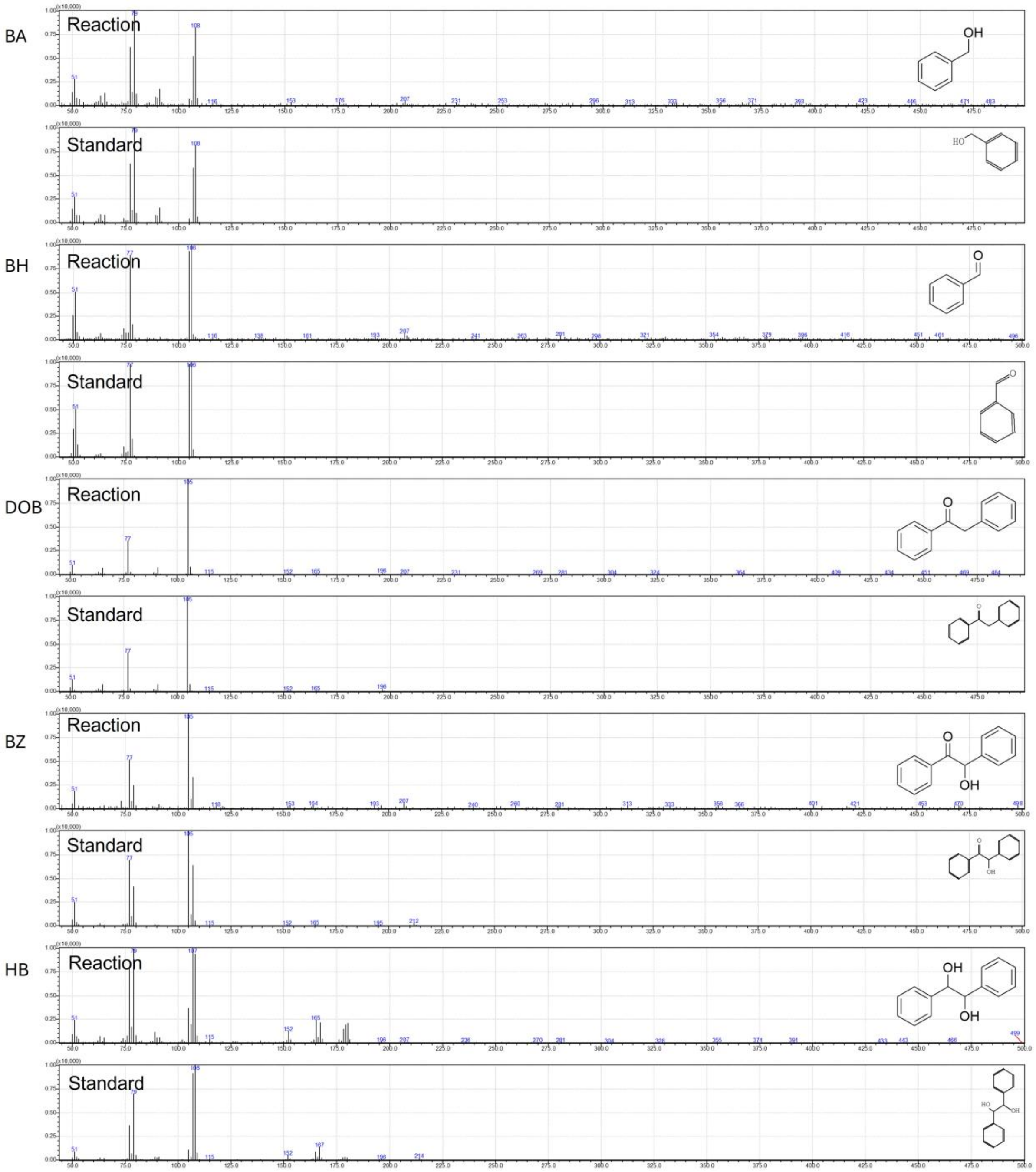

Figure S5. The standard and detected mass spectra of BA, BH, DOB, BZ, and HB. Reaction condition: BA is 10 mg, photocatalyst is 10 mg, CH_3CN is 5 mL, Ar is at 1 atm, visible light (0.35 W cm^{-2}).

GC-MS analysis was conducted to qualify the generated products from photocatalytic C-C coupling of BA. The results were compared with the standard mass spectra of these products from the GC-MS library. As shown in **Figure S5**, both the standard and detected mass spectra

of BA, BH, HB, DOB and BZ from GC-MS were observed and confirmed the generated products, including BH, HB, DOB and BZ.

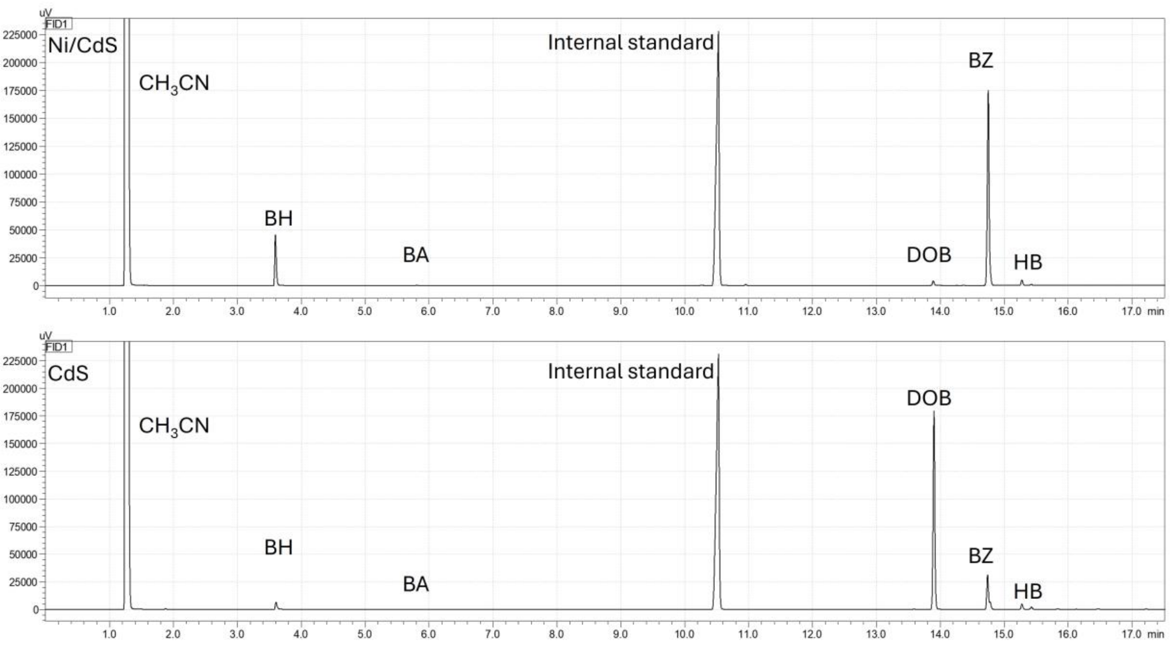

Figure S6. The GC spectra of reaction solutions using CdS after 9 h of visible light irradiation (bottom) and using 0.3% Ni/CdS after 5 h (upper). Reaction condition: BA is 10 mg, photocatalyst is 10 mg, CH_3CN is 5 mL, Ar is at 1 atm, visible light (0.35 W cm^{-2}).

GC analysis was conducted to quantify the generated products from photocatalytic C-C coupling of BA. As shown in **Figure S6**, GC spectra of reaction solution after using both photocatalysts display several peaks, which correspond to CH_3CN (reaction solvent), BH, BA, internal standard (ISTD), DOB, BZ and two peaks of HB. The ISTD (8 mg methylparaben) was added to calculate the quantitative analysis of BA and products. When CdS is used, the peak intensity of DOB is significantly higher than others, indicating that DOB is a main product using CdS after 9 h of visible light irradiation. In contrast, for 0.3% Ni/CdS, the peak intensity of BZ is significantly higher than other peaks, indicating that BZ is a main product using 0.3% Ni/CdS after 5 h.

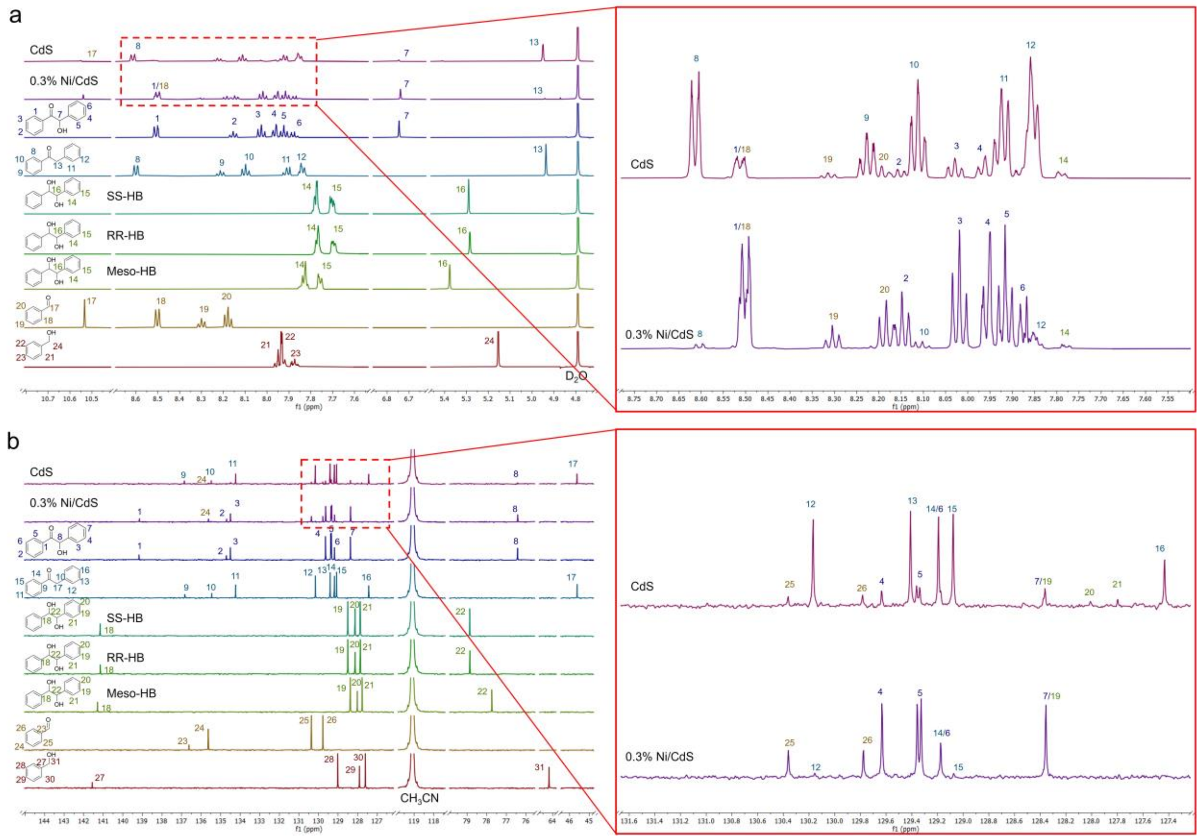

Figure S7. The (a) ^{1}H and (b) ^{13}C NMR spectra for BA, all generated products, and the reaction solutions when using 0.3% Ni/CdS after 5 h of visible light irradiation and using CdS after 9 h. Condition: For NMR analysis of standard chemicals, chemical is 5 mg, CH_3CN is 2.5 mL, D_2O is 2.5 mL; for NMR analysis of reaction solutions, reaction solution is 2.5 mL and D_2O is 2.5mL. Reaction condition: BA is 10 mg, photocatalyst is 10 mg, CH_3CN is 5 mL, Ar is at 1 atm, visible light (0.35 W cm^{-2}).

The ^{1}H and ^{13}C NMR analyses were conducted to further identify the generated products from conversion of BA. The products in reaction solutions were identified through comparing ^{1}H and ^{13}C NMR spectra of the reaction products with the standard compounds. As shown in **Figure S7**, all peaks in both ^{1}H and ^{13}C NMR spectra of the standard compounds were identified and labelled, and their corresponding chemical compounds clearly indicated in the

diagram. All peaks in both ^{1}H and ^{13}C NMR spectra of the reaction solution were also labelled and compared with the peaks of standard compounds to determine the specific products generated when using 0.3% Ni/CdS after 5 h of visible light irradiation and using CdS after 9 h. For 0.3% Ni/CdS, both ^{1}H and ^{13}C NMR spectra of the reaction solution primarily show peak for BZ, with smaller peaks for BH byproduct, DOB and HB. In contrast, for CdS, both ^{1}H and ^{13}C NMR spectra of the reaction solution primarily show peak for DOB, with smaller peaks for BZ, BH and HB. All results can match the GC spectra presented in **Figure S6**.

Table S2. Photocatalytic performance of photocatalysts for C-C coupling of BA to DOB and BZ in literature.

Ref	Catalyst	Reaction Condition	BA amount	Reaction time	Light Source	Conversion / Selectivity					AQY
						BA	BH	DOB	BZ	HB	
7	$Zn_{0.2}In_2S_{3.2}$ (10 mg)	1 mL CH_3CN	0.10 mmol	12 h	6 W blue LEDs (455 nm)	>99%	-	30%	61%	-	0.248% (BZ)
	$Zn_{0.6}In_2S_{3.6}$ (10 mg)		0.10 mmol	12 h		>99%	-	64%	16%	-	0.325% (DOB)
8	$ZnIn_2S_4$ (20 mg)	10 mL CH_3CN/H_2O (3:7 v/v) and 10mM acetic acid	10mM	6 h	8 W Blue LED (440–460 nm)	~100%	-	73%	~20%	~2%	0.563% (DOB)
		10 mL CH_3CN and 10mM acetic acid	10mM	18 h		~100%	-	15%	83%	-	0.171% (BZ)
9	$Zn_3In_2S_6$-STAB (20 mg)	5 mL CH_3CN	0.35 mmol	11h	300 W Xe lamp, visible light (λ > 400 nm)	99%	-	11.9%	30.8%	53.8%	0.107% (BZ)
2	$Zn_{0.6}Cd_{0.4}S$ (10 mg)	10mL CH_3CN/H_2O (3:7 v/v)	0.1 mmol	4h	300 W Xe lamp, visible light (λ > 420 nm)	77.14%	~16%	-	~2%	81.2%	2.84% (C-C coupled products)
This work	CdS (10mg)	5 mL CH_3CN	10 mg	9h	300 W Xe lamp, (420–780 nm, 0.35W/cm^2)	100%	2.8%	80.4%	14.6%	2.4%	4.42% (DOB)
	0.3% Ni/CdS (10mg)	5 mL CH_3CN	10 mg	5h		100%	15%	1.2%	81.5%	3.2%	3.01% (BZ)

It is important to compare the photocatalytic performance in producing DOB and BZ between previous studies and our results, to highlight the improvement of our photocatalytic system. **Table S2** provides detailed information, including the catalysts used, reaction conditions, amount of BA, reaction time, light source, conversion rate of BA, and selectivity of different products. The results indicate that our CdS provides better photocatalytic selectivity for DOB and 0.3% Ni/CdS provides better selectivity for BZ than the results presented in literature [2,7–9].

To better understand our reaction system, the apparent quantum yield (AQY) for generation of desirable products were calculated [1,2]. Based on equations (1) and (2) in **Supporting**

Information, in our reaction system, the calculated $AQY_{(BZ, 0.3\% Ni/CdS)}$ is 4.42% when using 0.3% Ni/CdS photocatalysts, and $AQY_{(DOB, CdS)}$ is 3.38% when using CdS photocatalysts. The previous studies on photocatalytic C-C coupling of BA to DOB or BZ did not calculate the AQY for generation of DOB and BZ by using their photocatalysts. The AQY for their work was calculated by using the published data in their papers (Please note that we used the light source intensity to substitute the light irradiation intensity). Results presented in **Table S2** demonstrated that our AQY is higher than other works, indicating the photon utilization efficiency of 0.3% Ni/CdS and CdS photocatalysts is higher than others.

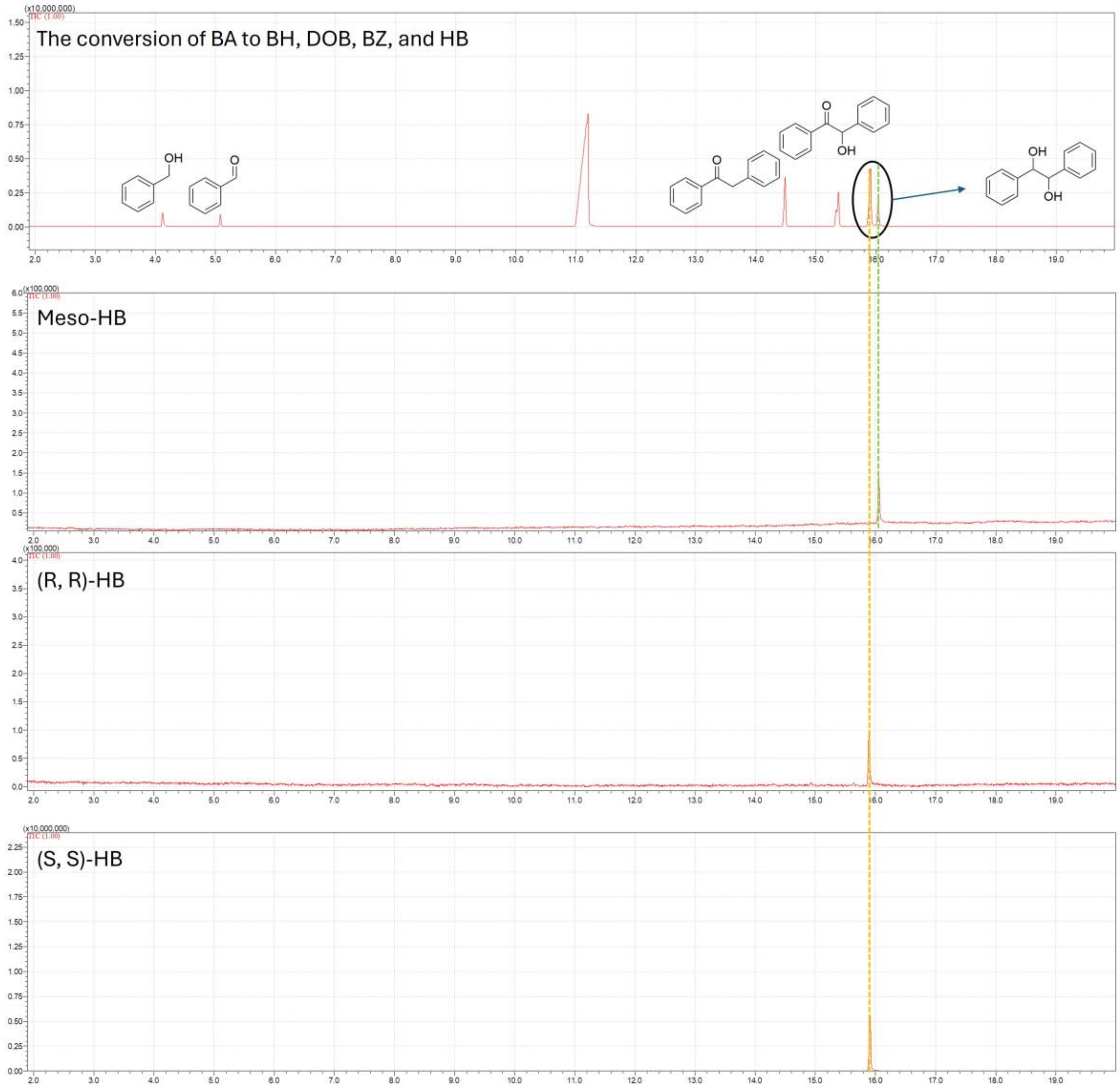

Figure S8. The GC-MS spectra of the products in conversion of BA, and the GC-MS spectra of meso-HB, (R, R)-HB, and (S, S)-HB.

The HB generated is an intermediate in the photocatalytic C-C coupling of BA to DOB or BZ. However, GC-MS spectrum of reaction solution reveals two distinct peaks for HB intermediates, which might be (R,R)-HB, (S,S)-HB, and meso-HB, with different chiral forms of HB. Therefore, we further analyzed these three standard HB forms using GC-MS to identify the specific HB products. As shown in **Figure S8**, the front HB peak represents (R,R)-HB or (S,S)-HB, while the back HB peak represents meso-HB.

Table S3. The photocatalytic performance in the conversion of different chiral HB to BH, BA, BZ, and DOB

Entry	Catalyst	Atmosphere	Reactant	Conversion / Selectivity				
				HB	BH	BA	BZ	DOB
1	0.3% Ni/CdS	Ar	10 mg meso-HB	100%	1%	-	85.7%	13.8%
2	0.3% Ni/CdS	Ar	10 mg RR-HB	100%	0.7%	-	87.5%	11.4%
3	0.3% Ni/CdS	Ar	10 mg SS-HB	100%	0.8%	-	87.2%	12.2%

Reaction condition: reactant is 10 mg, photocatalyst is 10 mg, CH_3CN is 5 mL, Ar is at 1 atm, visible light power is 0.35 W cm^{-2}, 3 h.

Table S4. The photocatalytic performance in the conversion of FA

Entry	Catalyst	Reaction time	Conversion / Selectivity				
			FA	Furfural	HF	Dehydrated-HF	Dehydrogenated-HF
1	CdS	12 h	68.5%	8.5%	86.7%	-	-
2	CdS	24 h	100%	10.3%	88.6%	-	-
3	0.3% Ni/CdS	6 h	89.7%	34.7%	54.6%	-	-
4	0.3% Ni/CdS	24 h	100%	33.8%	63.4%	-	-

Reaction condition: FA is 10 mg, photocatalyst is 10 mg, CH_3CN is 5 mL, Ar is at 1 atm, visible light power is 0.35 W cm^{-2}.

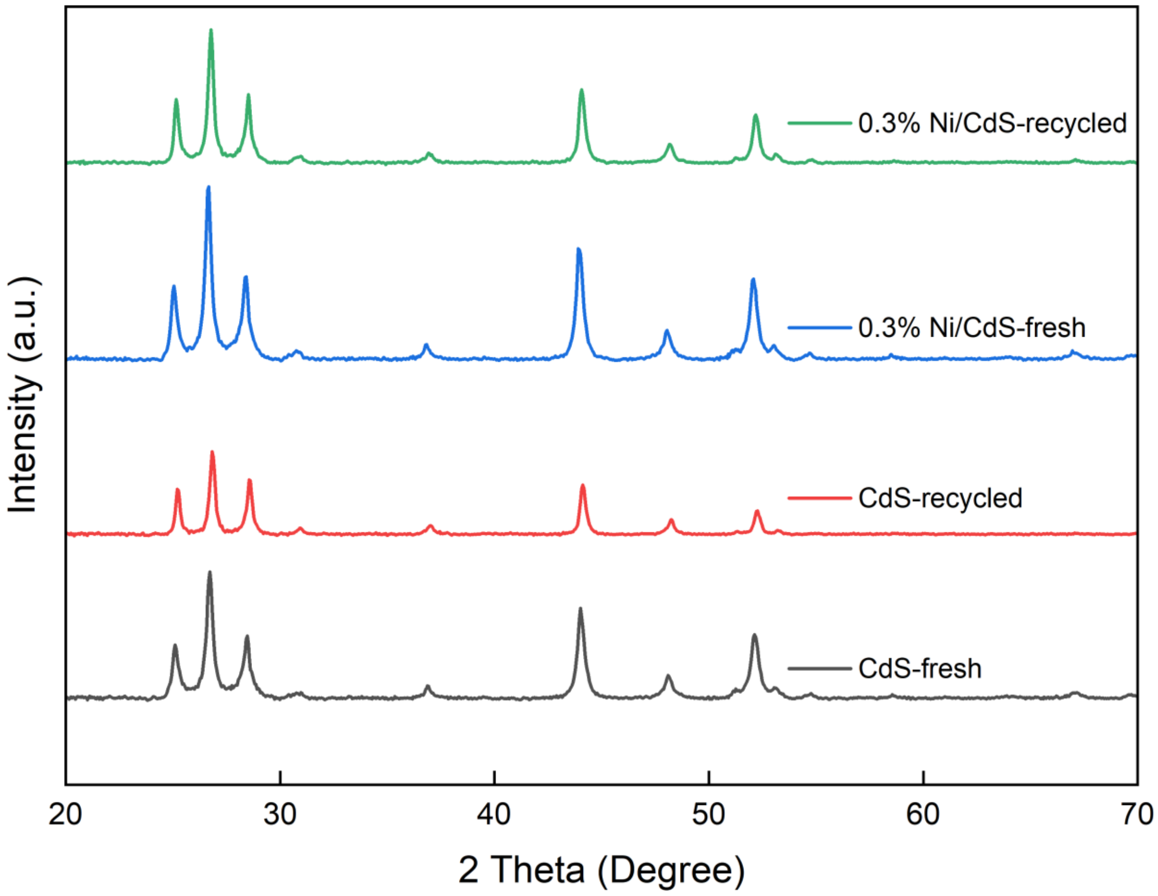

Figure S9. XRD patterns of fresh and recycled CdS and 0.3% Ni/CdS photocatalysts.

Photostability of photocatalysts is a crucial factor in photocatalytic reaction systems. XRD analysis was conducted to compare the structural integrity of CdS and 0.3% Ni/CdS photocatalysts before and after five cycles. As shown in **Figure S9**, the XRD patterns of both photocatalysts retain all their peaks before and after the 5 photocatalytic cycles, indicating that both photocatalysts exhibit high potential for long-term stability and durability in photocatalytic conversion of BA to DOB or BZ.

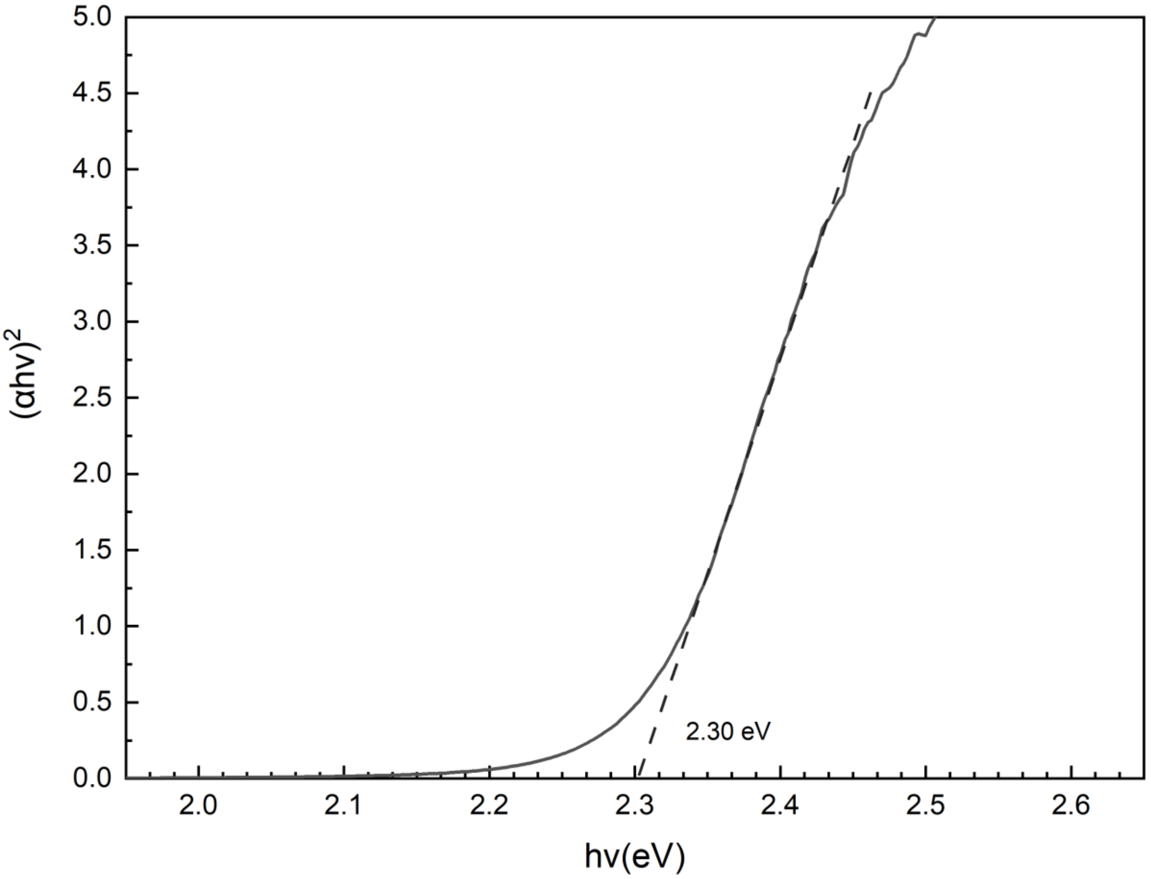

Figure S10. The $(\alpha h\nu)^2$ vs. hν curve obtained from DRS of CdS.

Based on the DRS results (**Figure 3a**), the bandgap energy (E_g) of CdS was calculated through the Kubelka-Munk function, defined as $(\alpha h\nu)^n = A(h\nu - E_g)$, where α, h, ν, n, and A are the absorption coefficient, Planck constant, light frequency, exponent, and an energy-independent constant, respectively. The exponent n depends on the type of bandgap in the semiconductor. For CdS photocatalysts, n is 2 in the Kubelka-Munk function, as it is a typical direct bandgap semiconductor. As shown in **Figure S10**, E_g of CdS is determined to be 2.30 eV. In addition, the E_g of x% Ni/CdS is equal to the E_g of CdS, as the introduction of Ni clusters on the surfaces of CdS cannot alter the crystal structure of CdS [10,11].

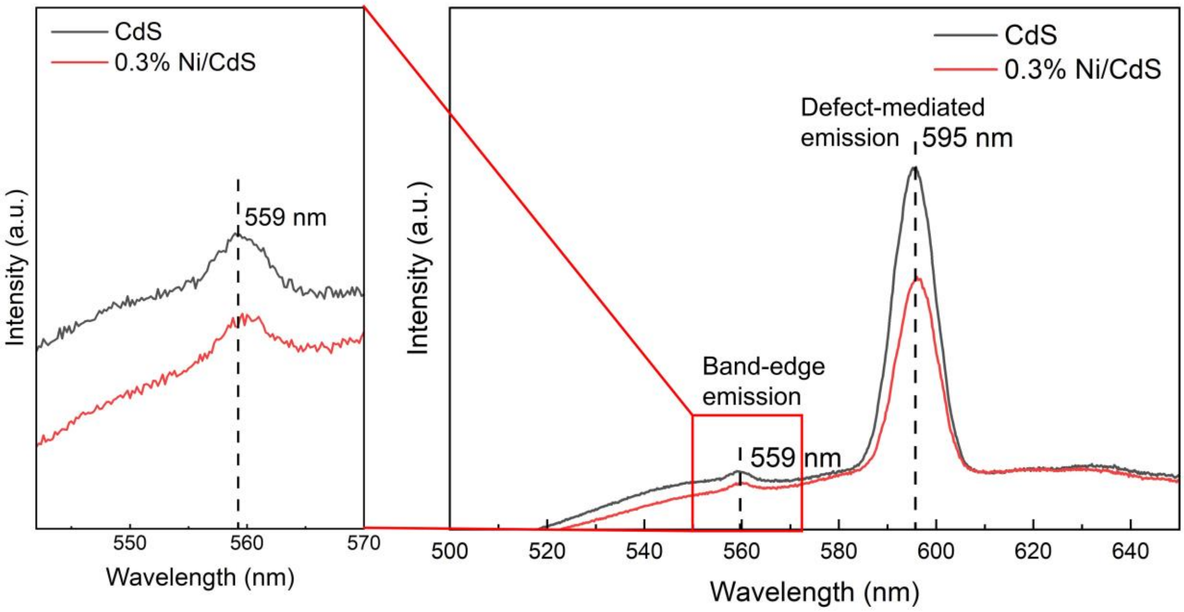

Figure S11. Powdery PL spectra of CdS and 0.3% Ni/CdS ($\lambda_{excitation}$ = 400 nm).

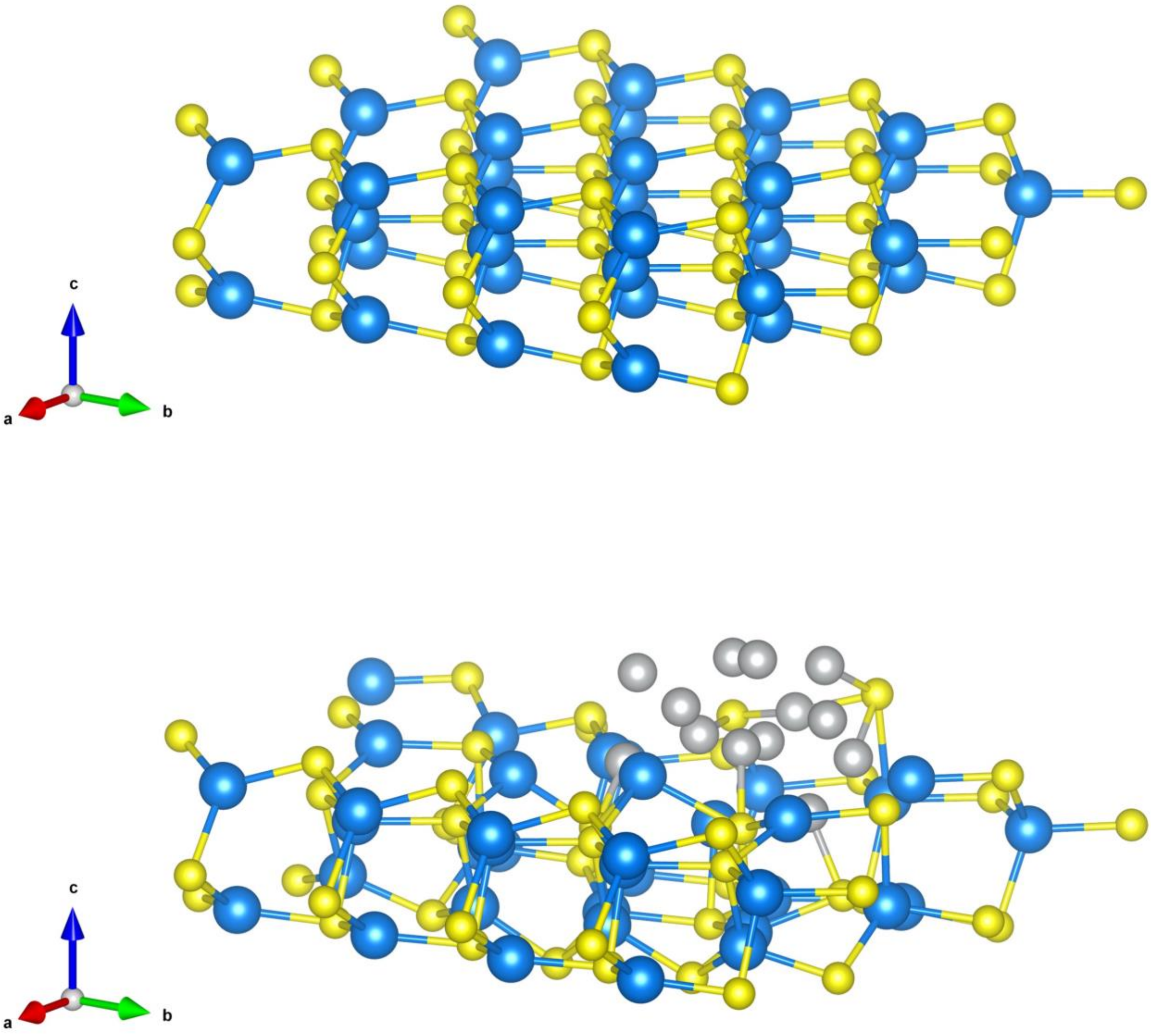

Figure S12. The created structures of CdS (upper) and Ni/CdS (bottom).

Table S5. Peak assignments for HB intermediates adsorption on CdS and 0.3% Ni/CdS photocatalysts.

Vibrational frequency (cm^{-1})	Vibrational mode	Assignation
3656	ν(O-H)	O-H bonds in HB
3540	ν(O-H)	H-bonded O-H bonds in HB on photocatalysts
3517		
3001	ν($C-H_3$)	C-H bonds in CH_3CN
2982		
2945		
2866	ν(C-H)	C-H bonds in phenyl and bridge moiety of HB
2844		
2825		
1200	ν(C-H)	C-H bonds in phenyl of HB
1135		
1055	ν($C-H_3$)	C-H bonds in CH_3CN
1032		
1009	ν(C-H)	C-H bonds in bridge moiety of HB
919	ν(C-O)	C-O bonds in HB
880	δ Ni–O(H)	Ni adsorbed O-H bonds in HB

Table S5 lists all peaks along with their potential vibrational modes and corresponding assignments from the time-resolved ATR spectra in **Figures 5e-f**. This table allows for easy identification of specific functional groups in different chemicals such as HB and CH_3CN solvent in our reaction system to analyze the adsorption modes of HB on photocatalyst surfaces. The detailed analysis of distinct characteristic peaks is discussed in **Section 3.6.2**.

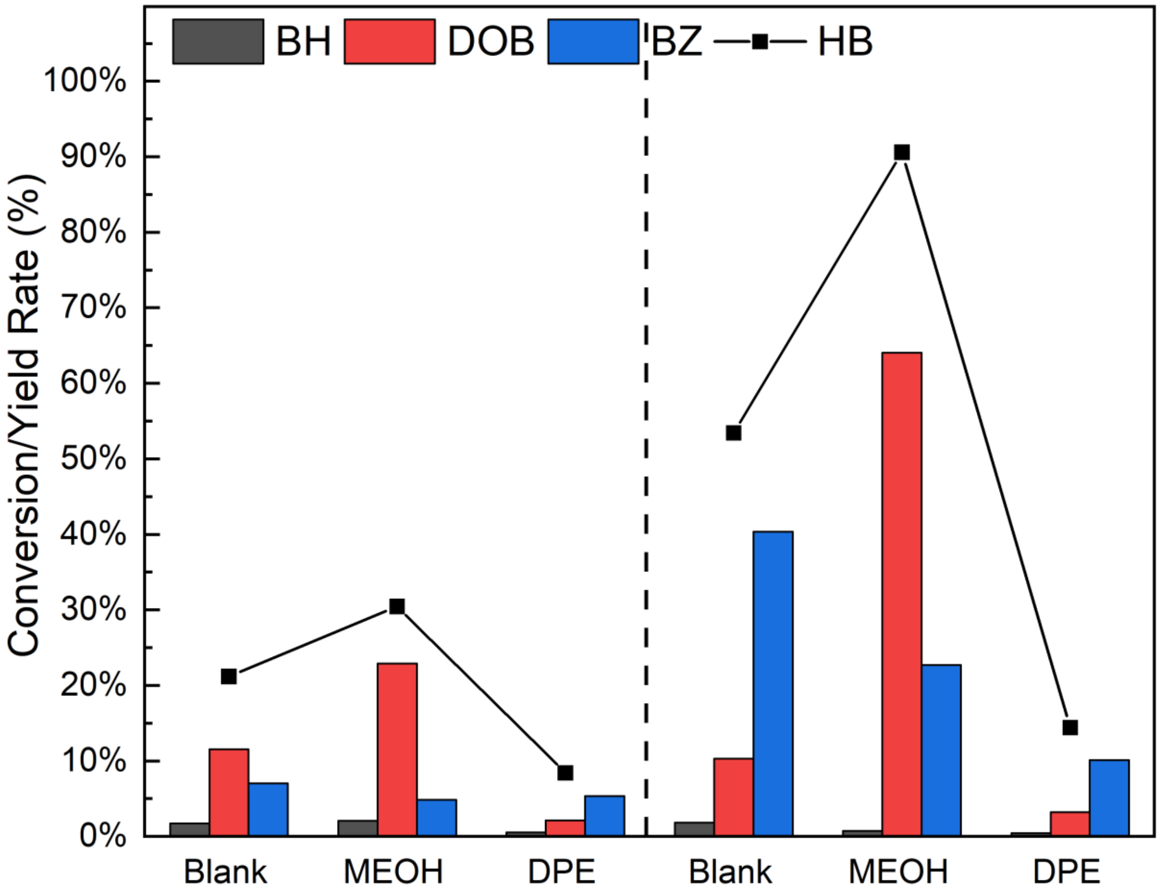

Figure S13. Conversion of HB intermediates to corresponding products with different scavengers using CdS and 0.3% Ni/CdS photocatalysts respectively. Reaction condition: HB is 10 mg, photocatalyst is 10 mg, CH_3CN is 5 mL, Ar is at 1 atm, visible light power is 0.35 W cm^{-2}, 20 min. O-centered radical scavengers: 30mg MEOH, C-centered radical scavengers: 30mg DPE.

Table S6. The calculated E_g, E_{CB} and E_{VB} of CdS and 0.3% Ni/CdS photocatalysts

Photocatalysts	E_g	E_{CB}	E_{VB}
CdS	2.30 eV	-1.13 eV	1.17 eV
0.3% Ni/CdS	2.30 eV	-0.57 eV	1.73 eV

The bandgap energy (E_g), conduction band edge (E_{CB}), and valence band edge (E_{VB}) are key factors to determine the photo-redox capabilities of both photocatalysts. E_g can be determined from the DRS results, as shown in **Figure S10**. The calculated E_g for both photocatalysts is 2.30 eV. Additionally, E_{VB} was obtained from the XPS-VB results. As shown in **Table S6**, E_{VB} of CdS is 1.17 eV, and the E_{VB} of 0.3% Ni/CdS is 1.73 eV. Using these values, E_{CB} can be calculated with the equation ($E_{CB} = E_{VB} - E_g$). As a result, E_{CB} of CdS is -1.13 eV, and E_{CB} of 0.3% Ni/CdS is -0.57 eV.

Reference

(1) Ma, M.; Wang, R.; Shi, L.; Li, R.; Huang, J.; Li, Z.; Li, P.; Konysheva, E. Y.; Li, Y.; Liu, G.; Xu, X. Defect- Expedited Photocarrier Separation in $Zn_2In_2S_5$ for High- Efficiency Photocatalytic C─C Coupling Synchronized with H_2 Liberation from Benzyl Alcohol. *Adv. Funct. Mater.* **2024**. https://doi.org/10.1002/adfm.202405922.

(2) Wang, R.; Zheng, Z.; Li, Z.; Xu, X. Photocatalytic C-C Coupling and H_2 Production with Tunable Selectivity Based on $Zn_xCd_{1-x}S$ Solid Solutions for Benzyl Alcohol Conversions under Visible Light. *Chem. Eng. J.* **2024**, *480*, 147970. https://doi.org/10.1016/j.cej.2023.147970.

(3) Kresse, G.; Furthmüller, J. Efficient Iterative Schemes for Ab Initio Total-Energy Calculations Using a Plane-Wave Basis Set. *Phys. Rev. B* **1996**, *54* (16), 11169.

(4) Perdew, J. P.; Burke, K.; Ernzerhof, M. Generalized Gradient Approximation Made Simple. *Phys. Rev. Lett.* **1996**, *77* (18), 3865.

(5) Hammer, B.; Hansen, L. B.; Nørskov, J. K. Improved Adsorption Energetics within Density-Functional Theory Using Revised Perdew-Burke-Ernzerhof Functionals. *Phys. Rev. B.* **1999**, *59* (11), 7413.

(6) Grimme, S. Semiempirical GGA- type Density Functional Constructed with a Long- range Dispersion Correction. *J. Comput. Chem.* **2006**, *27* (15), 1787–1799.

(7) Luo, N.; Hou, T.; Liu, S.; Zeng, B.; Lu, J.; Zhang, J.; Li, H.; Wang, F. Photocatalytic Coproduction of Deoxybenzoin and H_2 through Tandem Redox Reactions. *ACS Catal.* **2020**, *10* (1), 762–769. https://doi.org/10.1021/acscatal.9b03651.

(8) Han, G.; Liu, X.; Cao, Z.; Sun, Y. Photocatalytic Pinacol C-C Coupling and Jet Fuel Precursor Production on $ZnIn_2S_4$ nanosheets. *ACS Catal.* **2020**, *10* (16), 9346–9355.

https://doi.org/10.1021/acscatal.0c01715.

(9) Zhao, S.; Song, S.; You, Y.; Zhang, Y.; Luo, W.; Han, K.; Ding, T.; Tian, Y.; Li, X. Tuning Redox Ability of $Zn_3In_2S_6$ with Surfactant Modification for Highly Efficient and Selective Photocatalytic C-C Coupling. *Mol. Catal.* **2022**, *528*, 112429. https://doi.org/10.1016/j.mcat.2022.112429.

(10) Simon, T.; Bouchonville, N.; Berr, M. J.; Vaneski, A.; Adrovic, A.; Volbers, D.; Wyrwich, R.; Döblinger, M.; Susha, A. S.; Rogach, A. L.; Jäckel, F.; Stolarczyk, J. K.; Feldmann, J. Redox Shuttle Mechanism Enhances Photocatalytic H_2 Generation on Ni-Decorated CdS Nanorods. *Nat. Mater.* **2014**, *13* (11), 1013–1018. https://doi.org/10.1038/NMAT4049.

(11) Huang, Y.; Liu, C.; Li, M.; Li, H.; Li, Y.; Su, R.; Zhang, B. Photoimmobilized Ni Clusters Boost Photodehydrogenative Coupling of Amines to Imines via Enhanced Hydrogen Evolution Kinetics. *ACS Catal.* **2020**, *10* (6), 3904–3910. https://doi.org/10.1021/acscatal.0c00282.
